# Genetic and Functional Modifications Associated with Ovarian Cancer Cell Aggregation and Limited Culture Conditions

**DOI:** 10.3390/ijms241914867

**Published:** 2023-10-03

**Authors:** Joseph P. Grieco, Stephanie L. E. Compton, Grace N. Davis, Jack Guinan, Eva M. Schmelz

**Affiliations:** 1 Graduate Program in Translational Biology, Medicine, and Health, Virginia Tech, Blacksburg, VA 24061, USA; grieco@ohsu.edu; 2 Department of Human Nutrition, Foods and Exercise, Virginia Tech, Blacksburg, VA 24061, USA; stephanie.compton@pbrc.edu (S.L.E.C.); grace.davis@duke.edu (G.D.N.);

**Keywords:** ovarian cancer, aggregation, mitophagy, mitobiogenesis, hypoxia, glucose, metabolism, angiogenesis

## Abstract

The aggregation of cancer cells provides a survival signal for disseminating cancer cells; however, the underlying molecular mechanisms have yet to be elucidated. Using qPCR gene arrays, this study investigated the changes in cancer-specific genes as well as genes regulating mitochondrial quality control, metabolism, and oxidative stress in response to aggregation and hypoxia in our progressive ovarian cancer models representing slow- and fast-developing ovarian cancer. Aggregation increased the expression of anti-apoptotic, stemness, epithelial-mesenchymal transition (EMT), angiogenic, mitophagic, and reactive oxygen species (ROS) scavenging genes and functions, and decreased proliferation, apoptosis, metabolism, and mitochondrial content genes and functions. The incorporation of stromal vascular cells (SVF) from obese mice into the spheroids increased DNA repair and telomere regulatory genes that may represent a link between obesity and ovarian cancer risk. While glucose had no effect, glutamine was essential for aggregation and supported proliferation of the spheroid. In contrast, low glucose and hypoxic culture conditions delayed adhesion and outgrowth capacity of the spheroids independent of their phenotype, decreased mitochondrial mass and polarity, and induced a shift of mitochondrial dynamics towards mitophagy. However, these conditions did not reduce the appearance of polarized mitochondria at adhesion sites, suggesting that adhesion signals that either reversed mitochondrial fragmentation or induced mitobiogenesis can override the impact of low glucose and oxygen levels. Thus, the plasticity of the spheroids’ phenotype supports viability during dissemination, allows for the adaptation to changing conditions such as oxygen and nutrient availability. This may be critical for the development of an aggressive cancer phenotype and, therefore, could represent druggable targets for clinical interventions.

## 1. Introduction

Ovarian cancer is the 5th leading cause of cancer-related deaths in women, with an overall mortality rate of 50%; however, peritoneal dissemination decreases the survival rate of women with ovarian cancer to 31% [1]. The exfoliation of metastases can occur at any stage of the primary tumor’s development, contributing to the accumulation of a variety of genetic mutations and heterogenous advanced disease [2,3]. Exfoliated metastases can aggregate to form histologically heterogeneous spheroids. Regardless of their genotypic aberrations, aggregation aids in the survival of spheroids in the peritoneal cavity by promoting resistance to anoikis via dysregulation of oncogene and tumor suppressor signaling [4], resistance to drug treatment [5], and apoptosis [6], resulting in an increased metastatic potential [7]. These studies suggest that aggregation provides signals conducive for promotion of tumor cell survival during their transport in the hypoxic (1–2% O_2_) and glucose-starved peritoneal cavity [8] to their metastatic sites.

Spheroids are comprised of multiple tumor cells and recruited stromal cells forming compact three-dimensional structures with a proliferative outer layer, a quiescent interior, and often a necrotic core [9]. Factors promoting aggregation include expression of genes in TGFβ signaling, matrix protein expression and remodeling [7], integrin ligation by extracellular matrix molecules [10], and physical stresses such as fluid shear stress [11] that can activate mechanotransduction signaling pathways. Genome-wide and focused expression analyses and comparative transcriptomic studies have identified several genes and signaling pathways that are critical for the survival of the spheroids and the acquisition of drug resistance in a cell-type-dependent manner. For example, lung spheroids require changes in NRF2 and mTOR signaling [12], head-and-neck cancers show increased EGFR expression [13], while changes in ERBB2, SOX2, and E-cadherin expression are thought to be involved in the survival of stomach cancer spheroids [14]. Several reports have linked changes in TGFβ, β1-integrin, Hedgehog, STAT3, Nectin-4, or P70s6k expression [7,15,16,17,18] and others to the survival of ovarian cancer aggregates. Limitations of these approaches are routine culture conditions: 21% O_2_ and high concentrations of fetal calf serum and nutrients do not represent the conditions in the peritoneal cavity, which is characterized by low levels of oxygen, glucose, and other nutrients [8,19].

We have developed a murine ovarian surface epithelial (MOSE) cell model for progressive ovarian cancer comprised from benign cells, cells representing slow-developing (MOSE-L, causing lethal disease in ~100 days after injection of 1 × 10^6^ cells) and fast-developing (MOSE-L_TIC*v*_ lethal disease develops after 23 days after injection of 1 × 10^4^ cells) disease. The cell model is genetically and functionally comparable to the human disease [5,20,21] but this progression model avoids the influence of interindividual differences between cell lines on comparisons of different disease stages. We have previously reported that aggregation of murine ovarian cancer cells significantly altered mitochondrial morphology and function of murine serous ovarian cancer cells: Increased mitochondrial fragmentation during aggregation and hypoxia with little change in mitochondrial protein content per se [22] was associated with a reduced cellular respiration and spheroid growth [5]. Importantly, these adaptations were not lethal but reversible upon adhesion [23,24], suggesting that changes in genes regulating cell growth and mitochondrial dynamics and functions are critical during aggregation but plastic to adapt to changing environmental conditions.

While extensive analyses have been performed on solid tumors or metastases at distant sites, the changes in gene expression in disseminating ovarian metastases are less known. In the present study, we investigated the genetic and functional adaptations of ovarian cancer cells to aggregation in low-oxygen conditions to identify events that could be targeted for intervention studies aiming at reducing the viability of cancer aggregates and lower their capacity for a successful metastatic outgrowth. We determined genetic changes in cancer-related pathways and those associated with mitochondrial quality control regulation in response to aggregation and hypoxia, as well as the incorporation of cells harvested from the obese adipose tissue (stromal vascular fraction, SVF). We then investigated how the culture conditions differentially affect mitochondrial quality control balance and respiration in cells that represent slow-developing versus fast-developing disease to gain insight in the importance of mitochondrial morphology, dynamics, and function for the highly aggressive, stem-like phenotype. The results from this study suggest that the expression of tumorigenic regulators and mitochondrial adaptations impact the viability of ovarian cancer spheroids during dissemination. These effects were also more profound in our more aggressive MOSE-L_TIC*v*_ model than the slow-developing MOSE-L. These genetic and functional changes could provide new targets for preventive therapeutics to suppress cancer metastases and enhance the survival of women with ovarian cancer. Since aggregation-mediated enhanced survival is not unique to ovarian cancer metastases but have also been observed in disseminating colon and breast cancer aggregates [6], the results here may also be applicable to other cancers.

## 2. Results

### 2.1. Expression Levels of Cancer-Related Genes in Response to Aggregation and Hypoxic Media Conditions Correspond with the Aggressive Phenotype of MOSE Cells

**Figure 1:**
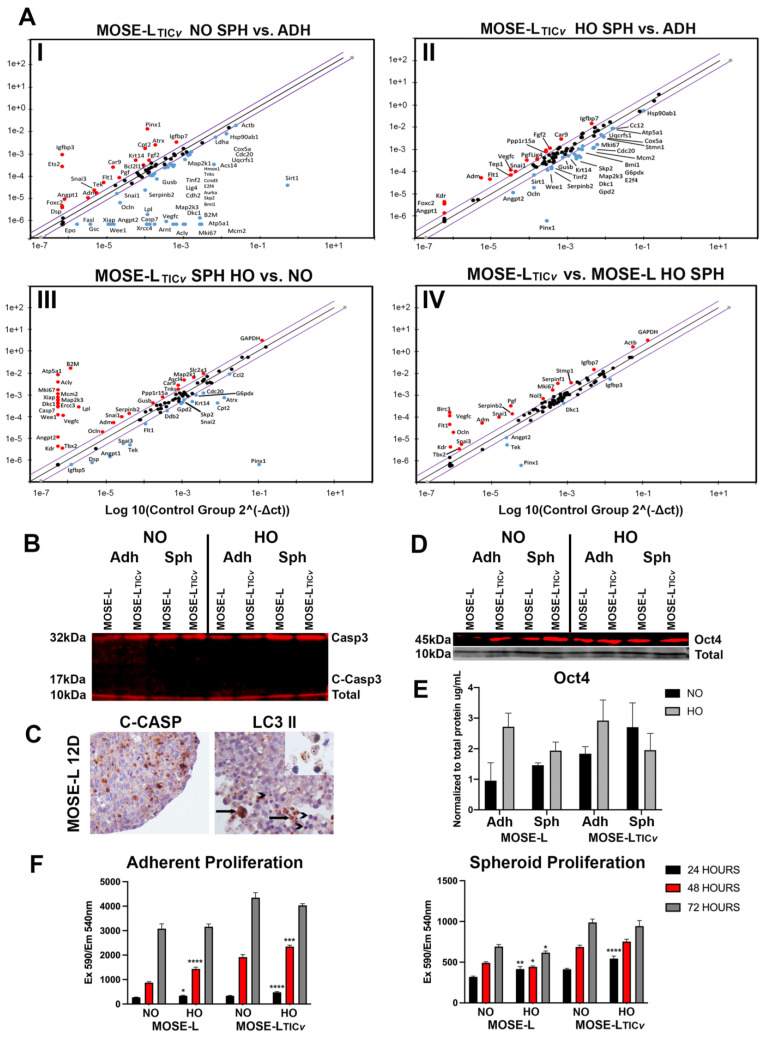
Genetic and functional changes in cancer-related pathways with during aggregation and in hypoxia. (**A**) RT-PCR analyses from the RT^2^ Profiler^TM^ PCR Array Mouse Cancer Pathway Finder. Data represented as individual comparing the response of MOSE-L or MOSE-L_TIC*v*_ cells and to aggregation and hypoxia (HO). (**AI**): MOSE-L_TIC*v*_ NO SPH vs. ADH. (**AII**): MOSE-L_TIC*v*_ HO SPH vs. ADH. (**AIII**): MOSE-L_TIC*v*_ HO SPH vs. NO SPH. (**AIV**): MOSE-L_TIC*v*_ HO SPH vs. MOSE-L HO SPH. Genetic changes were included if normalized ΔCT values were upregulated (red) or downregulated (blue) by a fold factor of 2 or higher. Equal cDNA from 3 pooled samples were used for the arrays. (**B**) Western blot analysis of Caspase-3 (Casp3) and cleaved Casp3 (C-Casp3) normalized to total protein control for adherent (ADH) cells or spheroid (SPH) MOSE-L and MOSE-L L_TIC*v*_ in normoxic/hypoxic (NO/HO) conditions. n = 3 biological replicates, analyzed by ANOVA. (**C**) IHC images of MOSE-L spheroids after 12 days incubated in optimal growth media immunostained for activated Casp3 and LC3B. Arrows are showing LC3 II positive cells undergoing autophagy, and arrowheads show apoptotic cells with aggregated nuclei (**D**) Western blot analysis of OCT4 protein expression. (**E**) Quantification of OCT4 protein expression normalized to total protein substrate using ImageJ. n = 3 biological replicates, analyzed by ANOVA (**F**) Adherent cells’ (Adh) and spheroids’ (Sph) proliferation rate was measured over 72 h comparing normoxic (NO) and hypoxic (HO) culture conditions for each timepoint using Alamar blue assay. * *p* < 0.05, ** *p* < 0.01, *** *p* < 0.001, **** *p* < 0.0001. n = 8–12 from 3 biological replicates, analyzed by ANOVA.

We first used qPCR arrays to quantify the expression levels of cancer-related genes in response to aggregation and hypoxia (Table S1A,B). In MOSE-L_TIC*v*_ spheroids, genes regulating angiogenesis (*Adm*, *Angpt1*, *Flt1*, *Fgf2*, *Fgf*, *Tek*), genes that can inhibit proliferation (*Pinx1*) or inhibit apoptosis (*Bcl2l1*) and increase cellular senescence (*Igfbp3* and *7*) were higher in spheroids than adherent cells. In contrast, genes involved in metabolism (*Acly*, *Acsl4*, *Atp5a1*, *Cox5a*, *Lpl),* angiogenesis-related genes (*Angpt2*, *Epo*, *Hmox1*, *Kdr),* apoptosis induction *(FasL*, *Casp7),* and genes regulating growth signaling pathways and cell cycle *(Map2k3*, *Map2k1*, *Cdc 20*, *Wee1)* were lower in spheroids (Figure 1(AI)). When cells were grown in hypoxia, aggregation altered the expression of genes in the same categories as seen in normoxic conditions albeit the magnitude appears to be lower (Figure 1(AII)). Some genes (e.g., *Ets2*, *Sirt1*, *Vegfc*) already responded to hypoxia in the adherent cells without additional change after aggregation.

The less aggressive MOSE-L cells also increased EMT genes and oncogene expression in normoxia (Supplemental Figure S1) and showed an upregulation in anti-apoptosis (*Bcl2l11*, *Nol3*, *Xiap*), angiogenesis (*Adm*, *Serpinf1*), and stemness (*Tbx2*, *Foxc2*) related genes but a downregulation in oncogenes (*Aurka*, *Cdc20*, *Bmi1*, *Ets2*, *Skp2*, *Tnks 1*,*2*), metabolism-related genes (*Acly*, *Atp5a1*, *Lpl*, *G6pdx*, *Gpd2*, *Cox5a*, *Uqcrfs1*), and apoptosis-regulating genes in hypoxic conditions (*Apaf1, Casp2*,*7*, *Fasl*) (Supplemental Figure S1). Comparing gene expression in spheroids grown in hypoxia versus normoxia, MOSE-L_TIC*v*_ spheroids showed a higher expression of genes promoting angiogenesis (*Adm*, *Angpt2*, *Kdr*, *Vegfc*), metabolism (*Acly*, *Acsl4*, *Atp5a1*, *Lpl*), apoptosis (*Casp7*, *Ppp1r15a*), EMT-related genes, and tumor suppressors (*Krtl4*, *Snai2*,3). We also found a lower expression of genes regulating vascular remodeling and angiogenesis, (*Angpt1*, *Flt1*, *Tek*), indicating that, while angiogenesis-promoting genes were upregulated during aggregation, hypoxia may lead to defects in vascular organization (Figure 1(AIII)).

To identify differences between the slow- and fast-developing disease, we compared gene expression levels in the MOSE-L and MOSE-L_TIC*v*_ spheroids and found that the MOSE-L_TIC*v*_ spheroids have higher levels of genes regulating angiogenesis, growth signaling and stem markers than the MOSE-L cells in both normoxic and hypoxic conditions (Figure 1(AIV), Supplemental Figure S1). Together, these data indicate that genes impacted by aggregation are downregulating metabolism, apoptosis, angiogenesis, and proliferation, but are upregulating EMT. The combination of aggregation and hypoxia affected the same functional categories but a differential response between the MOSE lines suggested that the aggressive phenotype is associated with a more robust response to culture conditions resulting in growth and apoptosis suppression as well as elevated EMT and angiogenesis.

### 2.2. Aggregation Supports Viability and Stemness but Suppresses Proliferative Functions

To link the alterations in gene expression levels in response to aggregation and hypoxia to functional changes, we next assessed the activation of cell death pathways, stemness, and proliferative capacity. As expected by the increase in anti-apoptotic genes, we did not observe the cleavage of caspase 3 in either adherent or spheroid MOSE cells after 48 h incubation in normoxic and hypoxic conditions (Figure 1B), indicating that neither aggregation nor hypoxia induced cell death via apoptosis. However, after 12 days of incubation in normoxic conditions, we observed cleaved caspase 3 as well as activated LC3 II and autophagosome formation, indicating that both pathways of cell death can be activated in the spheroids (Figure 1C).

As an important stemness marker for ovarian cancer [25], we determined changes in the protein levels of OCT4 (octamer-binding transcription factor 4). As shown in Figure 1D,E, there was a higher expression of OCT4 in the adherent and spheroid MOSE-L_TIC*v*_ in normoxic conditions that corresponds to their more aggressive, stem-like phenotype. OCT4 expression levels were elevated in both cell lines by aggregation in normoxic conditions; while both MOSE adherent cells exhibited increased OCT4 expression in hypoxia, there was little change in hypoxia in MOSE-L_TIC*v*_ spheroids (Figure 1D,E).

The growth rate of MOSE cells was significantly lower in spheroids at all observed timepoints (24–72 h) and oxygen conditions (*p* < 0.01 for MOSE-L at 24 h NO and MOSE-L_TIC*v*_ at 24 h NO; *p* < 0.0001 for all others) (Figure 1F). Further, both adherent and spheroid growth was higher in MOSE-L_TIC*v*_ than in MOSE-L cells (*p* < 0.01 at 24 h in adherent cells, *p* < 0.001 for spheroids for all conditions at all timepoints), confirming our previous results that spheroid formation led to a significantly reduced proliferation over time. The higher expression of genes regulating metabolism seen in the MOSE-L_TIC*v*_ spheroids compared to the MOSE-L spheroids may contribute to their greater proliferation rates observed even under hypoxic conditions and suggest the MOSE-L_TIC*v*_ spheroids have a higher capacity to adjust their genotype to aggregation to maintain viability and proliferation.

### 2.3. Incorporation of Obese SVF into MOSE Spheroids Changes Their Gene Expression

We have shown that obesity increases tumor burden in mice (Shea et al., in review Frontiers in Immunology). The SVF from adipose tissues can be recruited to tumors [26] and the incorporation of SVF harvested from obese adipose tissues into MOSE spheroids increased their invasive potential [5]. Here, we investigated changes in cancer-related genes in MOSE spheroids after incorporation of SVF at a ratio of 2:1 (MOSE cells to SVF) to determine genetic changes that promote an aggressive spheroid phenotype; this also may provide insight into the higher risk of ovarian cancer in women with obesity. Many genes were differentially expressed in the SVF and MOSE cells (Table S1B), leading to changes of gene expression levels in the heterogenous spheroids. Table 1 lists those genes that are higher or lower than expected by the mixture of cells or did not change despite differential expression in the MOSE cells or the SVF. In general, less genes were changed in the heterogenous MOSE-L than the MOSE-L_TIC_*_v_* spheroids in normoxia but more genes responded to hypoxic conditions in heterogenous MOSE-L spheroids.

The functional categories DNA repair, senescence, and telomere regulation and maintenance were overrepresented in genes changed in the heterogenous spheroids. DNA damage repair genes were upregulated in both cell lines by the incorporation of SVF (Table 1). This included the upregulation of *Errc* family members that regulate nucleotide excision repair as members of the TFIIH complex; this was independent of access to oxygen. *Atrx*, a gene that encodes a chromatin-modulating telomer stabilization protein [27] that has also been shown to be involved in DNA repair [28] and maintenance of genomic stability [29] was only upregulated in hypoxia while *Lig4*, encoding a DNA ligase involved in double-strand break repair was only elevated by SVF in normoxia. Interestingly, *Gadd45g* was reduced in the MOSE-L_TIC_*_v_* spheroids in both normoxia and hypoxia; a low expression via silencing correlates with a poor prognosis in AML [30] and a high expression represents a favorable prognostic marker in pancreatic and endometrial cancer (the human protein atlas, https://www.proteinatlas.org (accessed on 1 September 2023)). In addition to inducing apoptosis and differentiation as well as reducing colony formation in soft agar and chemosensitivity [29,30,31], *Gadd45g* overexpression suppressed DNA repair and disease progression, suggesting a function as tumor suppressor [30].

While aggregation did not alter many genes involved in telomere regulation and maintenance, several genes were upregulated especially in the MOSE-L heterogenous spheroids. This included *Sirt2*, *Tep1*, and *Tinf2*, all of which regulate telomerase expression and activity. A higher expression of *Dkc1* was observed in both MOSE spheroids. *Dkc1* encodes the dyskerin pseudouridine synthase 1, a protein that binds to the TERC complex and is essential for telomerase assembly and function. It is ubiquitously expressed and its overexpression in ovarian cancer has been associated with a poor outcome [32].

Aggregation itself increased the expression of genes regulating senescence but several genes (*Ets2*, *Igfbp3*) were more elevated in the heterogenous spheroids in both hypoxia and normoxia. Also upregulated were EMT-promoting genes in both cell lines. *Snai2*, a major regulator of EMT, and Cdh2, a mesenchymal marker, were upregulated in both normoxia and hypoxia by the SVF, indicating a push towards a mesenchymal phenotype in both cell types, while *Ocl* was only upregulated in heterogenous MOSE-L spheroids. In contrast, apoptosis regulators (*Apaf1*, *Casp9*) were only downregulated and metabolism regulators were only upregulated in heterogenous MOSE-L_TIC_*_v_* spheroids. Interestingly, the upregulation of *Acly*, *Acsl4*, *Gpd2* but downregulation of *Pfkl* suggests a shift in MOSE-L_TIC_*_v_* metabolism from glycolysis to other energy substrates. Increased fatty acid synthesis during progression was also observed in the MOSE model [33] and has been shown to be critical for tumor growth (see recent review [34]).

Cell cycle regulators were mostly upregulated in both cell types and oxygen conditions. *Aurka* was increased by the SVF in both cell types in hypoxia. In addition to mitosis regulation, *Aurka* has also been shown to be involved in DNA repair [35]. Another gene of interest was *Car9*, which was increased by aggregation in both oxygen conditions but was further elevated in the heterogenous spheroids of both cell types in hypoxia. *Car9* encodes carbonic anhydrase 9, an enzyme critical for extracellular acidification and matrix remodeling, thereby enhancing the metastatic potential of the tumor cells.

Thus, genes upregulated in spheroids by the SVF increase DNA repair, senescence, and telomere maintenance in the spheroids to support viability during anchorage-independent conditions, as well as increase EMT and stemness, promoting metastatic outgrowth with limited impact of the available oxygen levels.

**Figure 2:**
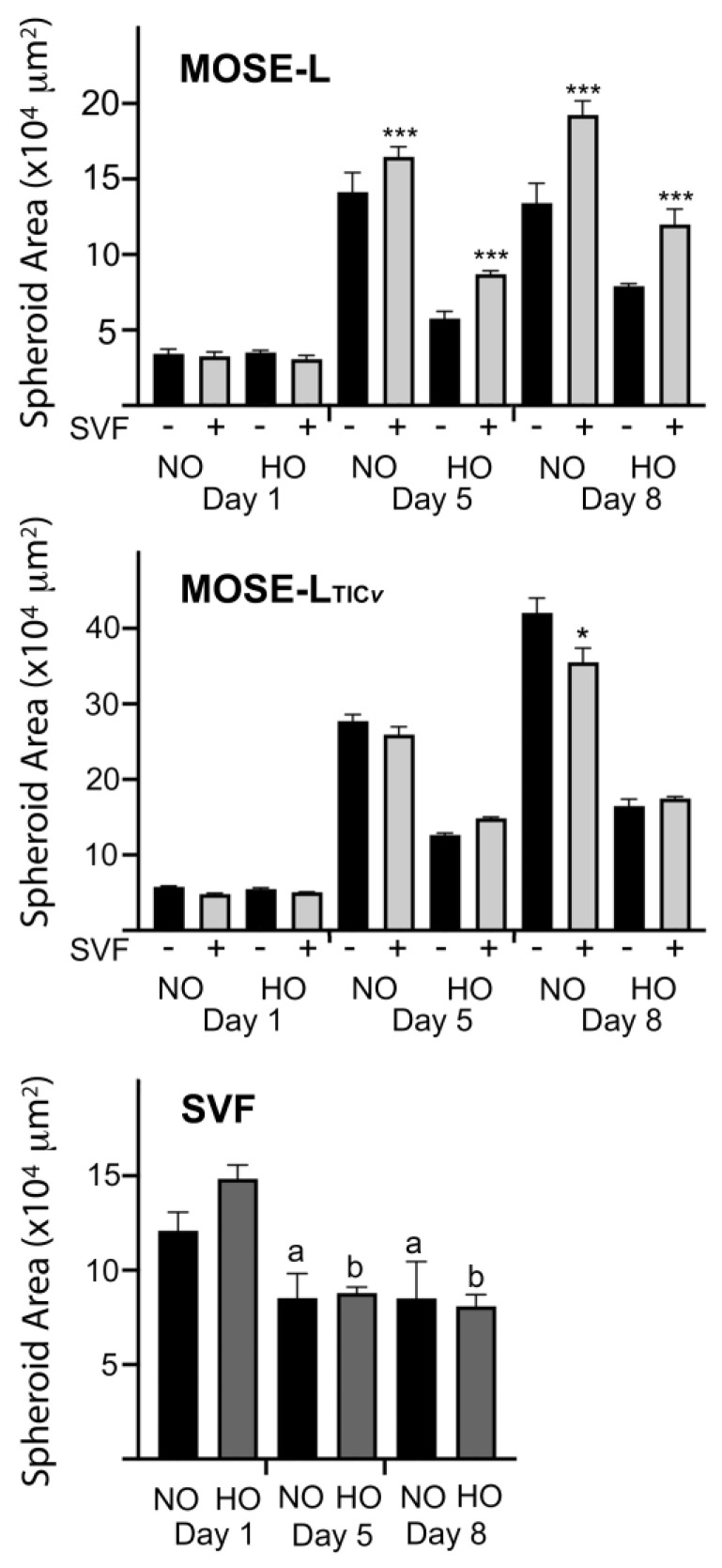
Changes in spheroid size after incorporation with obese SVF. The effect of the incorporation of the stromal vascular fraction (SVF) into MOSE spheroids at a ratio of 2:2 on spheroid size was measured by light microscopy using the Nikon NIS Elements^®^ software. * *p* < 0.05 and *** *p* < 0.001 vs. homogenous spheroids; “a” *p* < 0.01 and “b” *p* < 0.001 vs. the same conditions on day 1. Normoxia (NO), hypoxia (HO). n = 5–6 biological replicates, analyzed by ANOVA.

To determine if the SVF contributed to spheroid growth, we measured the spheroid area over time. As shown in Figure 2, the initial size of the spheroids was not affected by SVF on day 1 but significantly increased over time in normoxia but significantly less in hypoxia (*p* < 0.001 for both cell lines at day 5 and 8 compared to the previous timepoint). Only the MOSE-L spheroids were significantly larger after the incorporation of the SVF both in normoxic and hypoxic conditions. It is unclear if the increased heterogenous spheroid size is due to the growth of MOSE-L cells or the SVF as a result of the interaction of the different cell types. MOSE-L_TIC*v*_ spheroid size was not affected by the SVF and even appeared smaller after 8 days of incubation (*p* < 0.05) but was still larger than MOSE-L spheroids. We have shown previously that the SVF locates to the core of the MOSE spheroids and maintains viability [5]. The SVF alone formed small spheroids that did not grow and were significantly smaller after incubation for 5–8 days (*p* < 0.05 and *p* < 0.01 for normoxia and hypoxia, respectively) with no impact of hypoxia (Figure 2).

### 2.4. Changes in Mitochondrial Quality Control Genes Correlate with Increasing Malignancy, Aggregation, and Hypoxia

**Figure 3:**
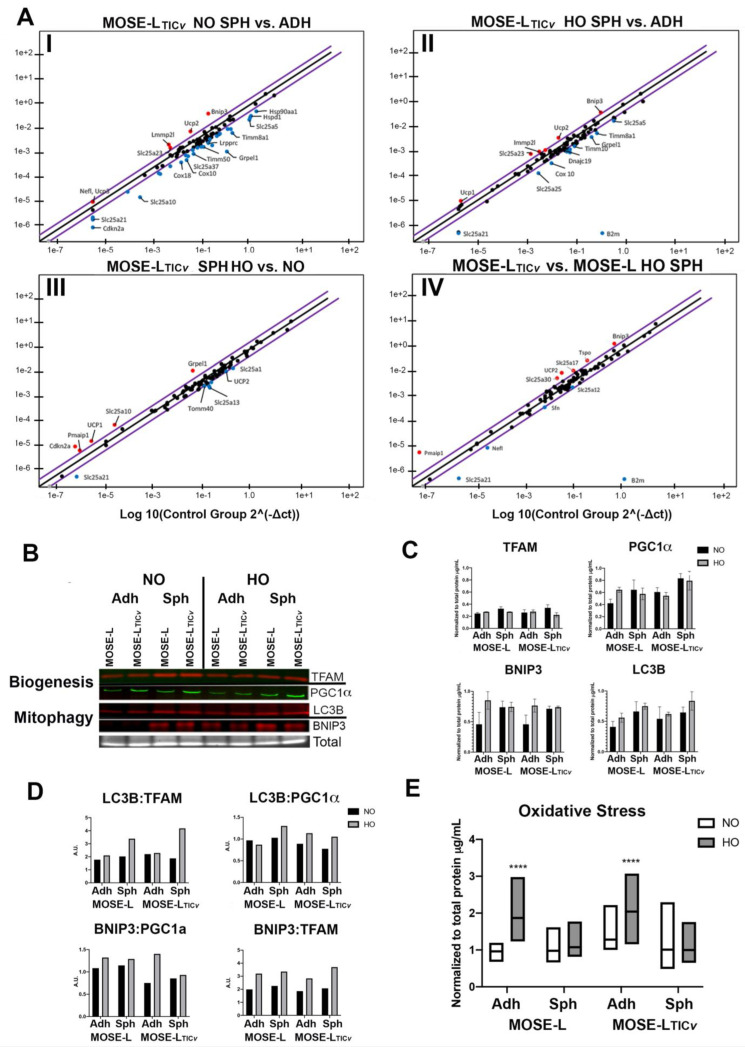
Genetic and functional changes in mitochondrial-related pathways with increasing malignancy and hypoxia. (**A**) RT-PCR analyses using the RT^2^ Profiler^TM^ PCR Array for Mouse Mitochondria (pooled cDNA from 3 biological replicates). (**AI**): MOSE-L_TIC*v*_ NO SPH vs. ADH. (**AII**): MOSE-L_TIC*v*_ HO SPH vs. ADH. (**AIII**): MOSE-L_TIC*v*_ HO SPH vs. NO SPH. (**AIV**): MOSE-L_TIC*v*_ HO SPH vs. MOSE-L HO SPH. Genetic changes were included if normalized ΔCT values were upregulated (red) or downregulated (blue) by a fold factor of 2 or higher. (**B**) Western blot analysis and quantification of mitophagy (BNIP3 and activated LC3B) and mitobiogenesis (PGC1α and TFAM) proteins in Adh/Sph MOSE-L and MOSE-L_TIC*v*_ cells in NO/HO media conditions. n = 3 biological replicates, analyzed by ANOVA. (**C**) Quantification of proteins was conducted using ImageJ. (**D**) Ratios of mitophagy proteins to mitobiogenesis proteins. (**E**) DCFDA quantification of ROS via plate reader analysis (485 ex/535 em) normalized to total protein. **** *p* < 0.0001. Adherent (Adh), spheroid (Sph), normoxia (NO), hypoxia (HO). n = 5–6 biological replicates, analyzed by ANOVA.

We have previously shown that changes in mitochondrial morphology during ovarian cancer progression are associated with a shift towards fusion and condensed mitochondrial morphology in adherent cells. Aggregation caused mitochondrial fragmentation but not cell death [22]. In this study, we aimed to identify genes that are affected by aggregation using RTPCR arrays for genes that are involved in mitochondrial transport, translocation, and apoptosis. Upon aggregation, genes regulating mitochondrial transport (*Immp21*, *Slc25a23*) and mitochondrial uncoupling (*Ucp1*,*2*,*3*) were higher expressed in the MOSE-L_TIC*v*_ in both normoxic and hypoxic conditions (Table S2); the key regulator of mitophagy *Bnip3* was also upregulated after aggregation. Compared to adherent cells, mitochondrial content-related genes that are localized in the inner and outer mitochondrial membranes and genes regulating mitochondrial transport were lower expressed in both hypoxic and normoxic spheroids. Further, genes regulating cytochrome c oxidase expression and maturation (C*ox10*, *Cox18*) were lower in hypoxic spheroids (Figure 3(AI,II)). A comparison between the expression of genes in spheroids grown in hypoxia versus normoxia showed a further upregulation of mitochondrial uncoupling (*Ucp2*,*3*), small molecule transport, and protein import by hypoxia (Figure 3(AIII)).

MOSE-L cells showed the downregulation of key regulators of metabolism (*Cox18*, *Cpt1b*) and mitobiogenesis/import (*Timm* and *Slc25a* family genes) after aggregation in normoxia. In contrast to MOSE-L_TIC*v*_ that were comparably resistant to hypoxia, MOSE-L responded to hypoxia with the downregulation of mitochondrial content genes (*Timm*, *Tomm*, *Immpl*, *Dnajc19*, and *Mtx2*) as well as pro-apoptotic genes (*Bak1*, *Pmaip1*) and tumor suppressors (*Cdkn2a*, *Trp53*). The key mitophagy-regulating gene, *Bnip3*, was upregulated in response to both aggregation and hypoxia (Figure S2). Comparing MOSE-L and MOSE-L_TIC*v*_ spheroids, *Bnip3* and uncoupling genes were higher expressed in the MOSE-L_TIC_*_v_* than in the MOSE-L spheroids in both normoxia and hypoxia (Figure S2) while several mitochondrial transporters and regulators of apoptosis were lower expressed in normoxia (Figure S2); only *Bnip3* and uncoupling genes were differentially expressed in hypoxia (Figure 3(AIV) and Figure S2). Together, these data suggest that gene changes with increasing malignancy, aggregation, and hypoxia decrease mitobiogenesis, apoptosis, proliferation, and metabolism, while mitophagy and uncoupling are increased with a more robust response in the aggressive MOSE-L_TIC_*_v_* in normoxia but a relative resistance to hypoxia.

### 2.5. Quantification of Mitochondrial Biogenesis and Mitophagy Changes in Response to Aggregation and Hypoxia

To correlate the genetic changes observed above (Figure 3(AI–AIV) and Figure S2) to mitochondrial functional adaptations, as a proof-of-concept we next quantified the expression of key hypoxia-regulated mitophagy (LC3B, BNIP3) and mitobiogenesis proteins (TFAM, PGC1α). As shown in Figure 3B,C, there was a differential response to aggregation and hypoxia between the MOSE lines. While spheroid formation increased the expression of PGC1α, BNIP3, and LC3B in both cell lines, hypoxia increased PGC1α and BNIP3 expression only in adherent cells, while there was a small increase in LCB3 only in MOSE-L spheroids. There was little change in TFAM expression levels in either cell line or culture condition (Figure 3B,C). Since the protein data were variable and the differences did not reach statistical significance, we next determined the ratio of mitophagy: mitobiogenesis to determine if one mechanism of quality control could be more apparent with increasing malignancy, aggregation, and hypoxic induction. As shown in Figure 3D, the ratio of mitophagy proteins over the mitobiogenesis proteins suggested a shift towards mitophagy with no drastic effect of aggregation but a higher ratio in hypoxia.

Hypoxia can promote the production of ROS, leading to highly depolarized mitochondria preceding mitophagy [36]. We thus determined how hypoxia and aggregation affect ROS production. There was a significant increase in ROS levels in adherent cells of both cell types by hypoxia (*p* < 0.001), confirming our previous results [22]. Aggregation itself did not increase ROS production but prevented hypoxia-induced ROS generation seen in the adherent cells (Figure 3E).

### 2.6. Determinants of Spheroid Formation and Viability

**Figure 4:**
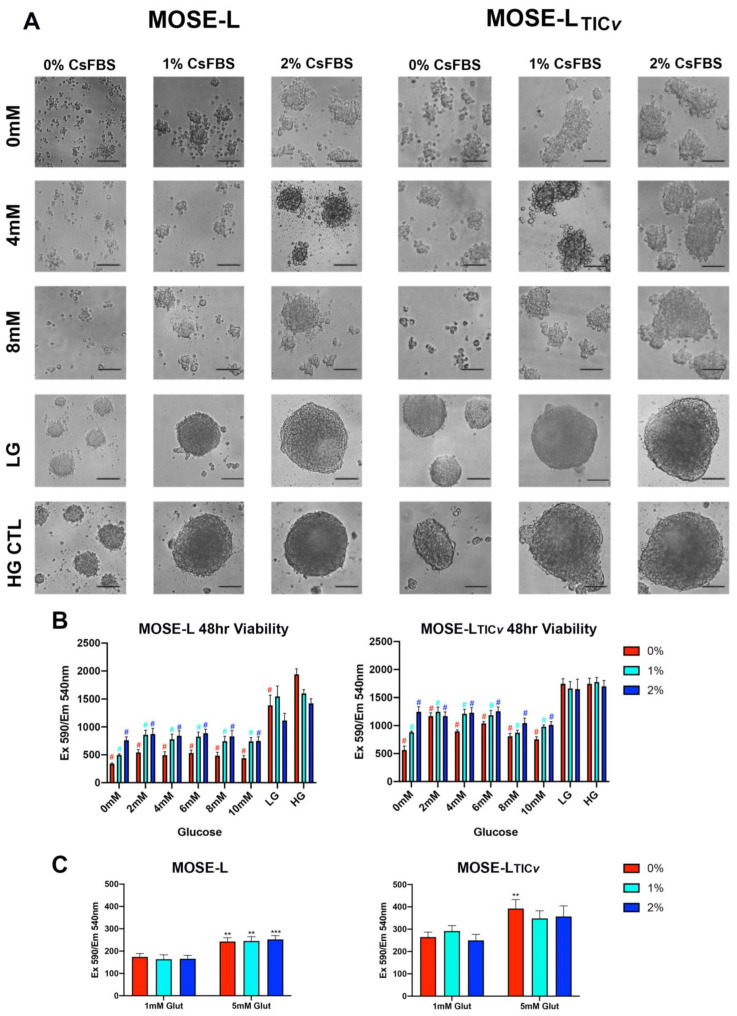
Spheroid formation capacity and viability in response to glucose, serum, and glutamine in hypoxia. (**A**) Representative images of MOSE-L and MOSE-L_TIC*v*_ cells incubated with DMEM without glucose supplemented with 0, 4, 8 mM glucose and 1 mM glutamine, low-glucose DMEM (LG—5 mM), or high-glucose DMEM (HG 27 mM) and 5 mM glutamine with charcoal-striped FBS (csFBS: 0, 1, 2%) for 24 h to determine their aggregation capacity. Scale bar set at 200 µm (**B**) Alamar blue assay of spheroids grown in different media conditions. # *p* < 0.05, colors represent comparisons made to HG control for each % growth serum. n = 5 biological replicates, analyzed by ANOVA. (**C**) Alamar blue assay of spheroids incubated in 6 mM glucose media with 1- or 5- mM glutamine for 48 h. n = 5–6 biological replicates, analyzed by ANOVA. ** *p* < 0.01, *** *p* < 0.001.

In addition to hypoxia, ovarian cancer cells are exposed to low levels of glucose during peritoneal dissemination [37]. We next investigated how glucose levels affect aggregation and proliferation of the MOSE cells when grown in hypoxic conditions. To limit the impact of the growth factors in FBS, we first incubated the cells without FBS. However, the lack of FBS induced cell death in adherent MOSE cells (data not shown); therefore, we added 0.5% charcoal-stripped serum (csFBS) to the medium that supports slow proliferation of adherent MOSE cells in both normoxia and hypoxia. As shown in Figure 4A, culturing cells in ultra-low adherence plates in hypoxia with medium containing no glucose or csFBS did not support aggregation of the MOSE-L cells and aggregation of the MOSE-L_TIC*v*_ cells was very limited. However, this formulation supported viability and slow proliferation of the cells for 48 h (Figure 4B) (see Figure S3 at 24 h), indicating that both cell types adjusted to these limited culture conditions without induction of cell death for at least 48 h. Increasing only csFBS concentrations improved aggregation in both cell types (Figure 4A), indicating that glucose is not critical for spheroid formation. However, increasing the glucose concentration to 2 mM significantly increased proliferation, whereas higher concentrations (up to 10 mM) did not further enhance proliferation (Figure 4B). Maximal growth stimulation was reached at 1% csFBS when the glucose concentration was at least 2 mM. In all conditions, MOSE-L_TIC*v*_ spheroids were larger and their proliferation rate higher than those of MOSE-L spheroids (*p* < 0.05 for all except 8 mM glucose in 1% csFBS). These results show that, while glucose levels are not necessary for the aggregation of the cancer cells and maintenance of their viability over 48 h, 2 mM of glucose is sufficient to support their proliferation in hypoxia over time.

Interestingly, culturing the MOSE cells in standard low- (5 mM) and high-glucose (25 mM) DMEM yielded viable, larger spheroids that increased in size with increasing concentrations of csFBS after 48 h incubation (Figure 4A, lower panels) and higher viability largely independent of the csFBS concentrations (Figure 4B and Figure S3B). Thus, media containing 5 or 25 mM of glucose but not the addition of glucose to the DMEM containing no glucose supported aggregation and significantly increased proliferation. The main difference between the two DMEM formulations beside the glucose levels are glutamine concentrations: the no-glucose DMEM contains 1 mM glutamine while the low- and high-glucose DMEM contain 5 mM glutamine. When we added glutamine to the medium containing no glucose, proliferation was significantly elevated after 24 h in MOSE-L cells independent of the csFBS concentrations (*p* < 0.01 for 0 and 1% csFBS and *p* < 0.001 for 2% csFBS); proliferation was also enhanced in the MOSE-L_TIC*v*_ spheroids, reaching statistical significance in the medium without csFBS (*p* < 0.01) (Figure 4C). Further, the viability was higher in the MOSE-L_TIC*v*_ spheroids than in the MOSE-L spheroids in all conditions (at least *p* < 0.05). Our results show that, while spheroids utilize glucose, glutamine is a limiting factor for aggregation and proliferation of MOSE cells. Importantly, the aggressive MOSE-L_TIC*v*_ spheroids were larger than those formed by the MOSE-L in all culture conditions, suggesting they can better adapt their survival mechanisms to the culture conditions or growth signals are less dependent on their microenvironment. How glutamine and other microenvironmental cues such as secreted matrix or adhesion proteins affect aggregation needs to be investigated in more detail.

### 2.7. Determinants of Adhesion and Outgrowth of Cancer Spheroids

**Figure 5:**
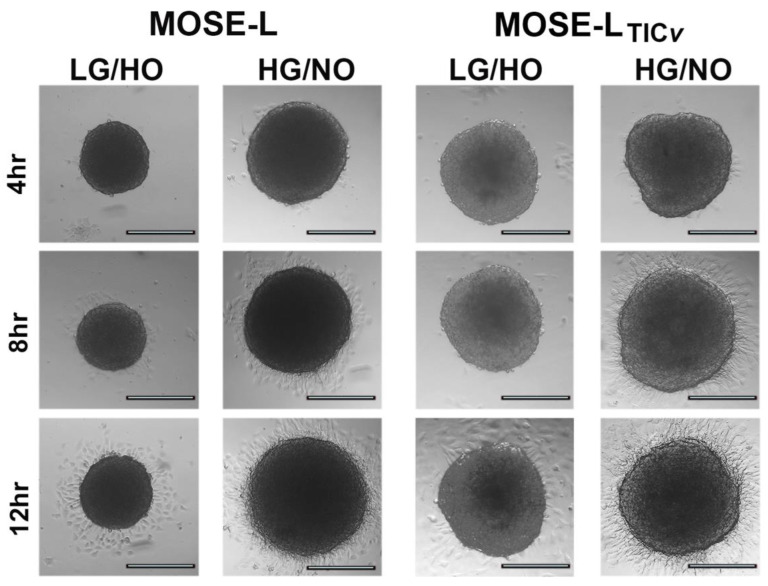
Adhesion capacity of spheroids in optimal vs. limited culture conditions. (A) Representative outgrowth images of MOSE-L and MOSE-L_TIC*v*_ spheroids incubated in optimal (HG/NO) and limited (LG/NO) culture conditions adhered for 4, 8, and 12 h. Scale bar set at 200 μm. Normoxia (NO), hypoxia (HO), low glucose (LG), high glucose (HG).

As a next step to aggregation and dissemination through the peritoneal cavity, spheroids need to adhere to and invade at secondary sites. Thus, we determined if limited access to nutrients and oxygen influenced the ability of the spheroids to adhere and promote secondary outgrowth using DMEM with 5 mM glucose (LG) and hypoxia (HO) representing limited culture conditions and DMEM with 25 mM glucose (HG) in normoxia (NO) representing optimal medium growth conditions; both media contained 5 mM glutamine. As depicted in Figure 5, all spheroids adhered to the tissue culture plates. However, the outgrowth—referring to cells that begin to grow out from the spheroid onto the culture dishes—was delayed when cultured in LG HO. Optimal culture conditions promoted a multi-layered outgrowth pattern in the MOSE-L_TIC*v*_ spheroid periphery; this was also observed in the MOSE-L spheroids, but the multilayered regions were much smaller. These data indicate that limited nutrients and oxygen levels did not affect the ability for spheroids to adhere but delayed subsequent outgrowth.

**Figure 6:**
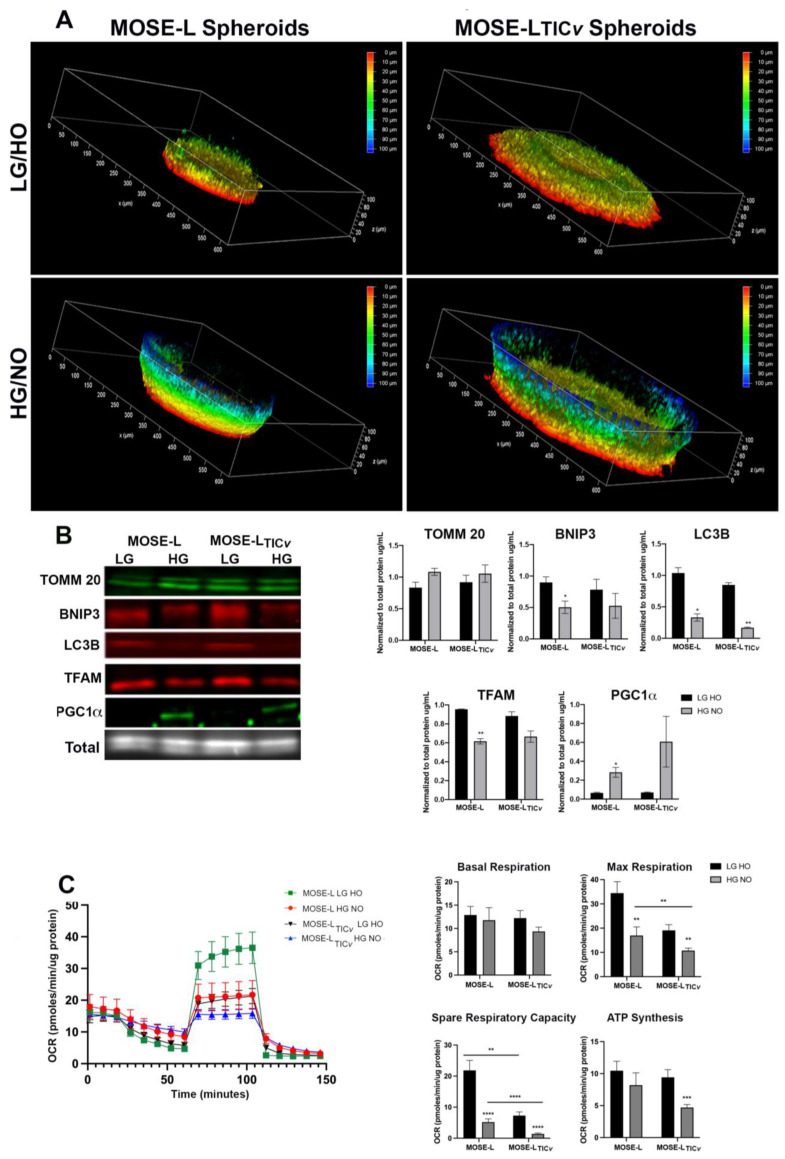
Mitochondrial localization and quality control in optimal vs. limited culture conditions. (**A**) Three-dimensional reconstructions of MitoTracker CMXRos–stained MOSE-L and MOSE-L_TIC*v*_ spheroids in optimal and limited culture conditions. The 100 µm z-stack reconstructions were depth coded to observe regional mitochondrial quantity and polarity. (**B**) Western blot analysis and quantification of mitophagy (BNIP3 and activated LC3B), mitochondrial content (TOMM20), and mitobiogenesis (PGC1α and TFAM) proteins in spheroids grown in optimal and limited culture conditions. N = 3 biological replicates. (**C**) Seahorse XFe96 oxygen consumption rate (OCR) and calculated basal and maximum respiration, ATP synthesis, and Spare Respiratory Capacity. N = 6–8 biological replicates. * *p* < 0.05, ** *p* < 0.01, *** *p* < 0.001, **** *p* < 0.0001.

Given the impact of the low-glucose medium and hypoxia on aggregation and adhesion, we next determined their impact on mitochondrial organization after short adhesion. As previously reported [22], highly polarized mitochondria were only visible in a thin layer of cells at the outside of the spheroids when the spheroids were grown in optimal medium without differences between the cell types beyond their different size (Figure 6A, bottom panels). Polarized mitochondria were prominent at the adhesion sites, spanning up to 50 μm into the spheroid in contrast to the single cell layer seen in non-adherent sites (spheroid sizes exceeded the limit for the confocal microscope). There was no difference in the thickness of cell layers containing polarized mitochondria between the cell types or culture conditions, suggesting that adhesion represents a signal for mitochondrial biogenesis, fusion, or polarization that is not dependent on glucose and oxygen availability.

Using Western blotting to determine changes in protein expression, we found that both mitophagy (BNIP3 (*p* < 0.05 for MOSE-L), LC3B (*p* < 0.05 for MOSE-L and *p* < 0.01 MOSE-L_TIC_*_v_*)) and mitobiogenesis regulators (TFAM (*p* < 0.05 for MOSE-L) were higher in limited conditions while PGC1α expression was lower (significant in MOSE-L *p* < 0.01). Mitochondrial content (TOMM20) was unchanged after 4 h of adhesion (Figure 6B). Whether the enhanced TFAM-dependent mitobiogenesis is the result of the culture conditions or of the adhesion signals needs to be investigated further. We then determined if these changes in mitochondrial regulators affect mitochondrial respiration during short-term adhesion. Overall, the oxygen consumption rate (OCR) was very low (Figure 6C), as we have shown previously [5]. There were no differences between culture conditions or cell types for basal respiration (Figure 6C, lower panels). However, maximum respiration was significantly higher in LG HO culture conditions in both MOSE-L and MOSE-L_TIC_*_v_* (*p* < 0.01) (Figure 6C, lower panels). Spare respiratory capacity (calculated as the difference between maximum and basal respiration) was also significantly higher in LG HO for both cell types (*p* < 0.0001). ATP synthesis only differed between culture conditions for MOSE-L_TIC_*_v_,* which was significantly higher in LG HO (*p* < 0.001). MOSE-L_TIC_*_v_* spheroids had a lower maximum respiration than the MOSE-L in HG NO (*p* < 0.01) and also had a lower spare respiratory capacity (*p* < 0.01 in LG HO; *p* < 0.0001 in HG NO).

## 3. Discussion

The survival of ovarian cancer cells exfoliated from the primary tumor requires the adaptation of the cells to their changing environment during disseminating through the peritoneal cavity. The aggregation of the cancer cells elicits such survival signals that allow for the modification of signaling pathways to prevent cell death due to anoikis, hypoxia, and lack of nutrients. However, these signals have yet to be elucidated in detail. Here, we investigated genetic and functional adaptations in cancer- and mitochondria-specific pathways that occur during cancer progression, aggregation, and hypoxic induction to mimic the peritoneal migratory environment. We aimed to better understand how aggregation contributes to spheroid survival and to discriminate the tumor-initiating phenotype of the MOSE-L_TIC_*_v_* cells from the less aggressive MOSE-L. We show that aggregation is associated with the upregulation of anti-apoptotic, stemness, and angiogenesis-related genes that can support the spheroid cells’ viability while genes involved in metabolism and proliferation were decreased, thereby lowering the metabolic demand when oxygen and glucose are not readily available.

The relative contribution of the functional categories identified above for the survival of the cell aggregates or their hierarchy is still unclear. However, upregulation of EMT genes that promote the generation of a mesenchymal stem-like phenotype and increasing stemness has been reported in aggregates from human ovarian cancer cell lines [38] and support their viability [39,40]. Stemness has been associated with a highly flexible metabolic phenotype, promoting self-renewal and high plasticity to overcome susceptibility of tumor microenvironmental stressors, including low oxygen or nutrient levels [41]. Further, stemness supports a senescent growth phenotype [42] with a low rate of glycolysis to suppress proliferation during circulation while maintaining ATP production in pancreatic cancers [43] while maintaining the viability of aggregates [40]. The ovarian stemness marker OCT (octamer-binding transcription factor 4) is highly expressed in stem-like cancer populations in the malignant ascites of ovarian cancer patients [25] and has been shown to contribute to a chemoresistant and aggressive ovarian cancer phenotype [44].

Increased EMT supports locomotive and metastatic capacity but also promotes invasion and immunological resistance through removal of epithelial markers and increased mesenchymal proteins [45,46]. Specifically, the upregulation of the stem markers *Tbx2* (a transcription factor that supports anchorage-independent growth [47] but also increased drug resistance [48]) and *Foxc2* (a transcription factor that also drives EMT [49]) in addition to the EMT markers *Krt14* and *Snai2,3* (increased in ovarian cancer spheroids and essential to spheroids’ attachment to and clearance of mesothelial cells during invasion [50,51] but also can suppress differentiation, promote stem cell transitions [52], and contribute to DNA repair [53]) by aggregation and hypoxia indicates that drivers of stemness and EMT are critical for the adjustment of proliferation, respiration, and adhesion of the ovarian cell aggregates. This, in conjunction with the observed increase in angiogenesis genes, including *Vegfc*, *Angpt*, and *Flt1*, aids in the ability for successful metastatic outgrowth especially with limited oxygen supply within the metastatic microenvironment. The more profound regulation of these genes can contribute to the more aggressive phenotype of the MOSE-L_TIC*v*_ spheroids.

We also observed a shift towards downregulation of mitochondrial content, biogenesis, and metabolism regulatory genes parallel to the upregulation of non-selective mitophagy regardless of oxygen content that was associated with a decreased oxidative stress. Mitochondria are important signaling organelles involved in proliferation, apoptosis, metabolism, oxidative stress removal, and more (14), and many of these genes were modified by aggregation (see above). In addition, key mitobiogenic and mitochondrial content genes—including the *Timm*, *Tomm*, and *Slc25a* family genes, important for translocating proteins encoded by nuclear DNA across the mitochondrial outer and inner membranes for oxidative phosphorylation [54]—were reduced in the spheroids. A decrease in these transport proteins can support cancer cell survival through forcing a reliance on glycolysis [55] as well as via reducing oxidative stress [29] and proliferation [56] but reducing apoptosis [57].

Aggregation of both MOSE-L and MOSE-L_TIC*v*_ cells increased the mitophagy-related gene, *Bnip3*, a hypoxia-activated key player in the process of removing highly depolarized mitochondria to limit internal oxidative stress. An increase in non-selective mitophagy through BNIP3 activation has been demonstrated in multiple cancers, including high-grade breast cancer [58] and non-small cell lung cancer [59]. Hypoxia increased BNIP3 in adherent cells to similar levels as seen in spheroids in normoxia but there was no further increase in hypoxic spheroids suggesting that physical or molecular signals of aggregation activate BNIP3 expression to a maximal level or aggregation prevented the response of spheroid cells to hypoxia. Interestingly, although we find the shift towards mitophagy with decreased TFAM expression at least two-fold lower than BNIP3 and activated LC3B, suggesting a low rate of mitobiogenesis (also supported by our gene expression data), PGC1α expression is relatively high especially in the MOSE-L_TIC*v*_ spheroids with little impact of hypoxia. PGC1α, a transcriptional co-activator and the master regulator of mitobiogenesis, is activated in hypoxia and activates mitobiogenesis and ROS clearance, thus aiding in survival and chemoresistance [60]. PGC1α also promotes anchorage-independent growth [61] and oxidative stress reduction via the SIRT1 axis [62]. Therefore, not only could the increase in BNIP3-driven mitophagy contribute to removing oxidative stress, but increased PGC1α expression could further support this antioxidant activity independently without enhancing mitochondrial content.

Abdominal obesity has been associated with an increased risk of developing and dying from ovarian cancer [63]. SVF incorporation from abdominal fatpads from obese mice into MOSE spheroids increased genes regulating DNA repair, senescence, and telomere regulation and maintenance. Interestingly, aggregation alone did not have a major effect on these functional categories while the progression of the MOSE cells showed an upregulation (*Aurka*, *Sirt1*, *Gpd2*, *Ocl*, *Ascl4*, *Snai1-3*) or downregulation (*Gadd45g*) of several genes in these categories (Table S1A), indicating that changes in cellular metabolism, DNA repair, and EMT occurred as a result of transformation and progression but were enhanced in the heterogenous spheroids.

DNA damage repair deficiency can lead to chromosome instability and accumulation of mutations, and it is tightly coordinated with cell cycle checkpoints, apoptosis, DNA replication or nucleotide metabolism to repair DNA damage before cell division. DNA damage repair has also been associated with drug and radiation resistance via repairing therapy-induced DNA damage and preventing apoptosis. Since DNA repair deficiency can sensitize tumors to platinum-based therapy [64], an increased DNA repair capacity may contribute to the drug resistance in the spheroids we observed previously [5]. Indeed, the elevated expression of genes involved in DNA repair have been corelated to a reduced overall survival [65]. While there were no differences found in mismatch repair genes between primary ovarian tumors and peritoneal metastases [66], genes involved in other types of DNA repair appear to be differentially regulated in metastases [65]. It is still unclear whether there are specific DNA repair mechanisms critical in ovarian spheroid survival.

A higher telomerase activity has been shown in spheroids from human ovarian cancer cell lines [67]. We found an upregulation of *Dkc1* expression in the heterogenous spheroids; DKC1 is essential for the assembly and activity of the telomerase complex. However, telomeres in cancer cells are in general short despite high telomerase expression. Thus, the contribution of DKC1 to tumorigenesis and an aggressive phenotype may not be due to the telomerase activity but other functions such as increasing cellular translation efficiency [68], proliferation, migration, and invasion [69]. This is supported by a low telomerase mutation rate in ovarian cancer that suggests that telomerase may be required to maintain the growth of a tumor [70]. Since many genes in the other categories are also involved in DNA repair (i.e., *Gadd45g*, *Snai1*), this function may be critical for the survival of the spheroids. Further, short telomeres can recruit DNA repair proteins and telomere maintenance requires components of the double strand DNA repair machinery [71], linking telomere lengthening to DNA repair.

Malignant ascites contains low levels of glucose (4.9 μM) and glutamine (0.3 μM), which are about half the concentrations observed in benign ascites [8]. Glucose was not required for the aggregation of the MOSE cells, and the proliferation of the spheroids was maximally stimulated with 2 mM glucose, which is lower than the levels found in commercially available media (5 mM glucose in low-glucose medium). Glucose starvation in hypoxia impeded the ability of ovarian cancer spheroids to adhere and delayed the outgrowth, but the underlying molecular mechanisms remain unclear. In contrast, glutamine was essential for aggregation and significantly supported proliferation, confirming its role as the preferred energy substrate in the MOSE spheroids [23]. Aggregation was also accompanied by a decrease in key metabolic genes associated with beta oxidation (*Cpt1b*) and electron transport chain (*Cox 10, 18*) as well as an increase in uncoupling genes (*Ucp2*, *3*) (Figure 2). This correlates well with the very low respiration in the MOSE spheroids; while there were differences in ATP synthesis and maximum respiration between the cell lines, the physiological relevance is unclear. However, we have recently shown that the spheroids can utilize other energy substrates and the most aggressive MOSE-L_TIC*v*_ spheroids exhibit the most flexible metabolism with little substrate specificity [72]. Since PGC1α also induces the expression of genes regulating fatty acid oxidation, gluconeogenesis, lipogenesis, and ATP production as an adaptation to nutrient availability or fasting and exercise, its increased expression in the spheroids suggests a role in the regulation of the metabolism in the spheroid cells in response to culture conditions.

Even the brief adhesion (4 h) provided a strong signal that caused the appearance of polarized mitochondria at the site of adhesion. Whether this is due to the fusion of the fragmented mitochondria or mitobiogenesis is still unclear since neither the qPCR arrays nor the Western blotting detected an increase in genes/proteins regulating these processes. This is likely due to an unresponsive majority of spheroid cells that were not receiving attachment signals and therefore did not undergo genetic and functional changes and effectively masked changes in mitobiogenesis or fusion genes or proteins in only the small number of adherent cells. The fact that low glucose and oxygen can delay adhesion and outgrowth but do not affect the change in mitochondrial morphology upon adhesion suggests that adhesion signals override the restrictions imparted by hypoxia and low glucose levels and confirm that mitochondrial morphology and functions are indeed plastic and reversible.

## 4. Materials and Methods

### 4.1. Cell Culture

From our MOSE cell system, we used MOSE-L cells, representing slow-developing disease, and MOSE-L_TIC*v*_ cells, representing fast-developing disease. These cells express fallopian tube markers and therefore likely represent the highly aggressive serous ovarian cancer [20]. The cells were routinely cultured in high glucose DMEM (28 mM), Sigma Aldrich, St. Louis, MO, USA) with 4% fetal bovine serum (FBS) (Atlanta Biological, Flowery Branch, GA, USA), 3.7 g/L sodium bicarbonate, 1% penicillin-streptomycin (termed “optimal growth medium”) in 5% CO_2_ and either normoxic (21% O_2_) or hypoxic (1–2% O_2_) conditions. Limited conditions consisted of low glucose DMEM (5 mM), (Sigma Aldrich, St. Louis, MO, USA), 1% charcoal-stripped FBS (csFBS), and 1–2% O_2_, representing oxygen levels in the malignant ascites [19]. Single or multiple aggregated cancer spheroids were generated in ultra-low adherence plates (Corning, Tewksbury, MA, USA) for 24–72 h as described [5].

### 4.2. Stromal Vascular Fraction Isolation

The stromal vascular fraction (SVF) of abdominal fat pads were collected as described previously [72,73,74]. Briefly, female C57BL/6 mice (7 weeks old, The Jackson Laboratoriy, Ellsworth, ME, USA) were fed a high-fat diet (60% kcal from fat—5% soybean oil and 55% lard—D12492, Research Diets, New Brunswick, NJ, USA). After 6 months on the diet, mice were sacrificed by CO_2_ asphyxiation, and the abdominal adipose tissues were harvested and digested with a collagenase IV. The harvested cells from 30 mice were then combined and grown in high glucose DMEM with 5% FBS and 10 μg/mL ciprofloxacin for 3 days. All animal procedures were conducted in accordance with the guidelines of the Virginia Tech Institutional Animal Care and Usage Committee (IACUC) under the protocol 21-030, reviewed and approved by the IACUC on 8/11/2-21.

### 4.3. Reverse Transcription Polymerase Chain Reaction

RNA was isolated from adherent and aggregated MOSE cells after incubation for 48 h utilizing the RNeasy mini kit (Qiagen, Germantown, MD, USA); cDNA synthesis was performed with the RT^2^ first strand kit (Qiagen) and pooled from three independent samples. The expression levels of cancer- or mitochondria-related genes were determined in duplicate using RT^2^ Profiler^TM^ PCR Arrays for Mouse Mitochondria (PAMM-087Z, Qiagen) or Mouse Cancer Pathway Finder (PAMM-033Z, Qiagen) on the ViiA7 Real-Time PCR system (Applied Biosystems, Waltham, MA, USA). Since some of the provided housekeeping genes changed during progression or in response to hypoxia, cancer gene cycle time (CT) values were normalized to *Terf2ip* and mitochondrial gene CT values were normalized to *Gapdh*. Gene expression was considered up- or downregulated if ΔCT changes were >2. Data analyses were performed with the Qiagen online tools. 

### 4.4. Monitoring Spheroid Proliferation and Size

To determine proliferation, MOSE-L and MOSE-L_TIC*v*_ were grown for 24, 48, and 72 h in the indicated culture conditions. Adherent monolayers were seeded in 96-well flat-bottom cell culture plates at a density of 5 × 10^3^ cells per well and incubated with alamarBlue (Invitrogen, Carlsbad, CA, USA) 4 h prior to absorbance reading. Spheroids were seeded at 5 × 10^3^ cells per well in round-bottom ultra-low adherence 96-well cell culture plates (Corning) and centrifuged at 900 RPM for 5 min to allow for the formation of a single spheroid. Spheroids were incubated with alamarBlue cell viability reagent 12 h prior to absorbance reading to allow for permeation into the aggregates. Plates were read at 600/570 nm to determine cellular proliferation via the ratio of oxidation: reduction of alamarBlue reagent indicative of viable cells. To monitor individual spheroids, images were taken with an inverted Nikon microscope. Spheroid sizes were calculated with the Nikon NIS Elements® 5.42.03 acquisition and quantitation software.

### 4.5. Monitoring of Spheroid Assembly and Outgrowth

The aggregation of cells to spheroids was observed qualitatively at 20× magnification on a Nikon inverted light microscope in ultra-low adherence plates. Spheroid outgrowth was determined after plating on glass-bottom 96-well plates (Corning) for 4, 8, and 12 h by imaging on an inverted Leica DmiL microscope at 10× magnification. Adhesion capacity was depicted through promotion of outgrowth protruding from the primary spheroids.

### 4.6. Protein Determination via Western Blotting

Adherent and aggregated MOSE cells were cultured for 48 h with the indicated treatments and were subsequently lysed in radioimmunoprecipitation buffer supplemented with protease and phosphatase inhibitors (ThermoFisher Scientific, Waltham, MA, USA). Protein concentrations were determined using the Pierce Bicinchoninic acid assay (BCA, ThermoFisher Scientific). After gel electrophoresis, proteins were transferred onto a PVDF membrane (BioRad, Hercules, CA, USA) and blocked with either 5% milk or BSA in 1X TBST. Primary antibodies directed against BNIP3 (Abcam, Waltham, MA, USA, ), CASP3 (Cell Signaling, Danvers, MA, USA), LC3B (Cell Signaling), OCT4 (Invitrogen), PGC1α (Millipore, Burlington, MA, USA), TOMM20 (Millipore), and TFAM (Abcam) and secondary IRDye 680 cw and 800 cw conjugated antibodies (Licor, Lincoln, NE, USA) were used for imaging with the Licor Odyssey CLx imager. Quantification was conducted with ImageJ, normalizing all proteins to total protein substrate (ThermoFisher Scientific) and data are presented as mean ± SEM with at least 3 biological replicates.

### 4.7. Immunohistochemistry

MOSE-L cells were seeded at a density of 0.5 × 10^5^ cells per well in ultralow-adhesion dishes (Corning). Culture medium was changed every 3 days. After 12 days in culture, the spheroids were harvested, gently centrifuged, rinsed in cold PBS and fixed in 10% buffered formalin, embedded into paraffin, and sectioned at 4–5 µm for immunohistochemical analyses of LC3 (Abgent, San Diego, CA, USA) and activated Caspase-3 (Cell Signaling) as described [75]. Images were taken using the Nikon Eclipse 80*i* microscope equipped with a DS-Fi1 Digital Camera and Nikon NIS Elements^®^ 5.42.03 acquisition and quantitation software. All images were processed with Adobe Photoshop Elements® 20.0.

### 4.8. Reactive Oxygen Species (ROS) Assay

Adherent cells were seeded at 1.5 × 10^4^ cells/well for 48 h in flat glass-bottom 96-well plates (Corning). Spheroids were formed in ultra-low adherent plates for 48 h and transferred to flat 96-well plates and washed with 0.25 mM sodium phosphate solution. After staining with 25 µM 2′7′-dichlorofluorescin diacetate (DCFDA) (Abcam) in Krebs–Ringer phosphate buffer for 45 min at 37 °C, internal ROS was quantified using a TECAN plate reader (485 ex/535 em). ROS production was normalized to total protein using BCA analysis.

### 4.9. Confocal Microscopy

Cultured MOSE cells were stained with 50 nM Mitotracker deep red (647 nm emission) prior to aggregation for 15 min followed by 2 × 5min washes with 1× PBS, and spheroids were subsequently generated as described above. Single spheroids were placed onto 35 mm glass-bottom dishes (Cellvis, Mountain View, CA, USA) for 4–8 h and then fixed with 100% methanol. To identify mitochondrial localization and prevalence, optical Z-stack slices were taken at 25× magnification using a Leica DMI8 MP inverted confocal microscope. Three-dimensional reconstruction of the spheroids was conducted using compatible LASx analysis software 2.0 to identify regional mitochondrial localization within each spheroid. Additional surface plots were generated to identify planar localizations of mitochondria.

### 4.10. Seahorse XFe96 Analysis

Mitochondrial respiration was determined using the XFe96 extracellular flux analyzer (Agilent, Santa Clara, CA, USA) as previously described [23]. Single spheroids were formed in 96-well ultra-low adherence plates at 1.5 × 10^4^ density for 24 h under the indicated conditions. Single spheroids were transferred to a XFe96 cell culture plate 4 h before the assay to allow for slight adherence of spheroids to the assay plate to avoid disturbance by the injections of inhibitors. Prior to the assay, the medium was changed to serum-free, bicarbonate-free medium. Experiments consisted of 3 min mixing, 2 min wait, and 3 min measurement cycles. Oxygen consumption rate (OCR) was measured under basal conditions and upon addition of mitochondrial inhibitors oligomycin (1.0 μmol/L), carbonylcyanide-p- trifluoromethoxyphenylhydrazone FCCP (3.0 μmol/L), and a combination of rotenone and antimycin A (1.0 μmol/L). These conditions were determined in preliminary optimization studies to achieve reproducible metabolic responses in 3D culture. All experiments were performed at 37 °C.

### 4.11. Statistical Analyses

Data are presented as mean ± SEM with biological replicates of at least n = 3. Malignant progression comparisons were conducted using ANOVA or individual student *t*-tests. Normoxic and hypoxic sample comparisons were also made using student-*t*-tests. Direct comparisons were made between optimal and limited media conditions as well as comparisons between MOSE-L and MOSE-L_TIC*v*_ spheroids using unpaired student *t*-tests. Results were considered significant at *p* < 0.05.

## 5. Conclusions

This study illustrates that the interplay of multiple cancer-related pathways—including proliferation, apoptosis, stemness, adhesion, and metabolism- with mitochondrial quality control, metabolic flexibility, and ROS production contribute to the survival of the aggregated MOSE cells even in limited culture conditions with low glucose and oxygen. Incorporating obese SVF mostly affected DNA repair and telomerase maintenance, with a great functional overlap of functions of genes in other categories, suggesting that DNA repair has a central role in the survival of ovarian spheroids during dissemination. We show that aggregation and viability of spheroids requires glutamine more than glucose, growth factors, and oxygen; however, the lack of glucose and oxygen delays the adhesion and the outgrowth of the spheroids, but not the appearance of polarized mitochondria at adhesion sites. Lastly, the differential response of the MOSE cells representing slow- and fast-developing disease indicates that these pathways are important for the development of an aggressive cancer phenotype. Deciphering the underlying signaling pathways that occur within these key pathways during metastasis from the primary tumor to secondary metastatic sites will provide new targets for therapeutics to suppress secondary adhesion and outgrowth of ovarian cancer metastases.

## Figures and Tables

**Table 1:** Gene changes by the addition of obese SVF into MOSE spheroids.

	Normoxia		Hypoxia	
MOSE-L + SVF	Lower expression	Higher expression	Lower expression	Higher expression
	*Cox5a*, *Pfkl*	*Ccnd2*, *Errc2*, *Errc3*, *Ets2*, *Ldha*, *Lig4*, *Ocl*, *Snai2*		*Atp5a1*, *Atrx*, *Ccnd2*, *Dsp*, *Errc3*, *Errc5*, *Gsc*, *Map2k1*, *Mki67*, *Ocl*, *Skp2*, *Snai2*, *Tnks*, *Wee1*
	Differential gene expression in MOSE cells and SVF but no change in gene expression in the heterogenous spheroids, suggesting change of expression in either cell type			
	Lower expression	Higher expression	Lower expression	Higher expression
	*Hmox1*	*Aurka*, *Cdh2*, *Igfbp3*, *Sirt2*, *Skp2*, *Stmn1*, *Tep1*, *Tinf1*	*Hmox1*	*Acly*, *Aurka*, *Bmi*, *Car9*, *Casp7*, *Cdh2*, *Dkc1*, *Ets2*, *Gadd45g*, *Igfbp3*, *Tinf2*
MOSE-L_TICv_ + SVF	Normoxia		Hypoxia	
	Lower expression	Higher expression	Lower expression	Higher expression
	*Angpt1*, *Apaf1*, *Amt*, *Atrx*, *Casp9*, *Ccnd3*, *Cpt2*, *Dsp*, *Fgf2*, *Flt1*, *Ing1*, *Ldha*, *Pfkl*, *Pgf*, *Pinx1*, *Tep1*	*Acly*, *Acsl4*, *Atp5a1*, *Bmi*, *Casp2*, *Casp7*, *Ccnd2, Dkc1*, *Errc3*, *Ets2*, *Gpd2*, *Lig4*, *Mcm2*, *Ppp1rl15a*, *Snai2*, *Tnks*, *Vegfc*	*Bmi1*, *Cox5a*, *E2f4*, *Snai1*, *Tbx2*	*Atrx*, *Bcl2l11*, *Car9*, *Ccnd2*, *Dkc1*, *Errc5*, *Gpd2*, *Igfbp3*, *Igfbp5*, *Skp2*, *Mki67*, *Snai2*
	Differential gene expression in MOSE cells and SVF but no change in gene expression in the heterogenous spheroids, suggesting change of expression in either cell type			
	Lower expression	Higher expression	Lower expression	Higher expression
	*Gadd45g*, *Gsc*, *Igfbp7*, *Sox10*, *Tek*		*Adm*, *Angpt1*, *Apaf1*, *Flt1*, *Gadd45g*, *Snai3*	*Atp5a1*, *Aurka*, *Cdc20*, *Cdh2*, *Mcm2*, *Mki67*, *Pinx1*

## Data Availability

Collected data from graphs and analysis can be requested and made available directly through contact of the corresponding author.

## Supplementary Materials

The following supporting information can be downloaded at: https://www.mdpi.com/article/10.3390/ijms241914867/s1.
