# Supplementary material for: Genetic and Functional Modifications Associated with Ovarian Cancer Cell Aggregation and Limited Culture Conditions
Source: Int J Mol Sci. 2023 Oct 3;24(19):14867. doi: 10.3390/ijms241914867 (PMC10573375; doi:10.3390/ijms241914867)
Supplement: Supplementary file 1 [file ijms-24-14867-s001.zip › ijms-2617132-supplementary.pdf]

Supplemental Figure S1

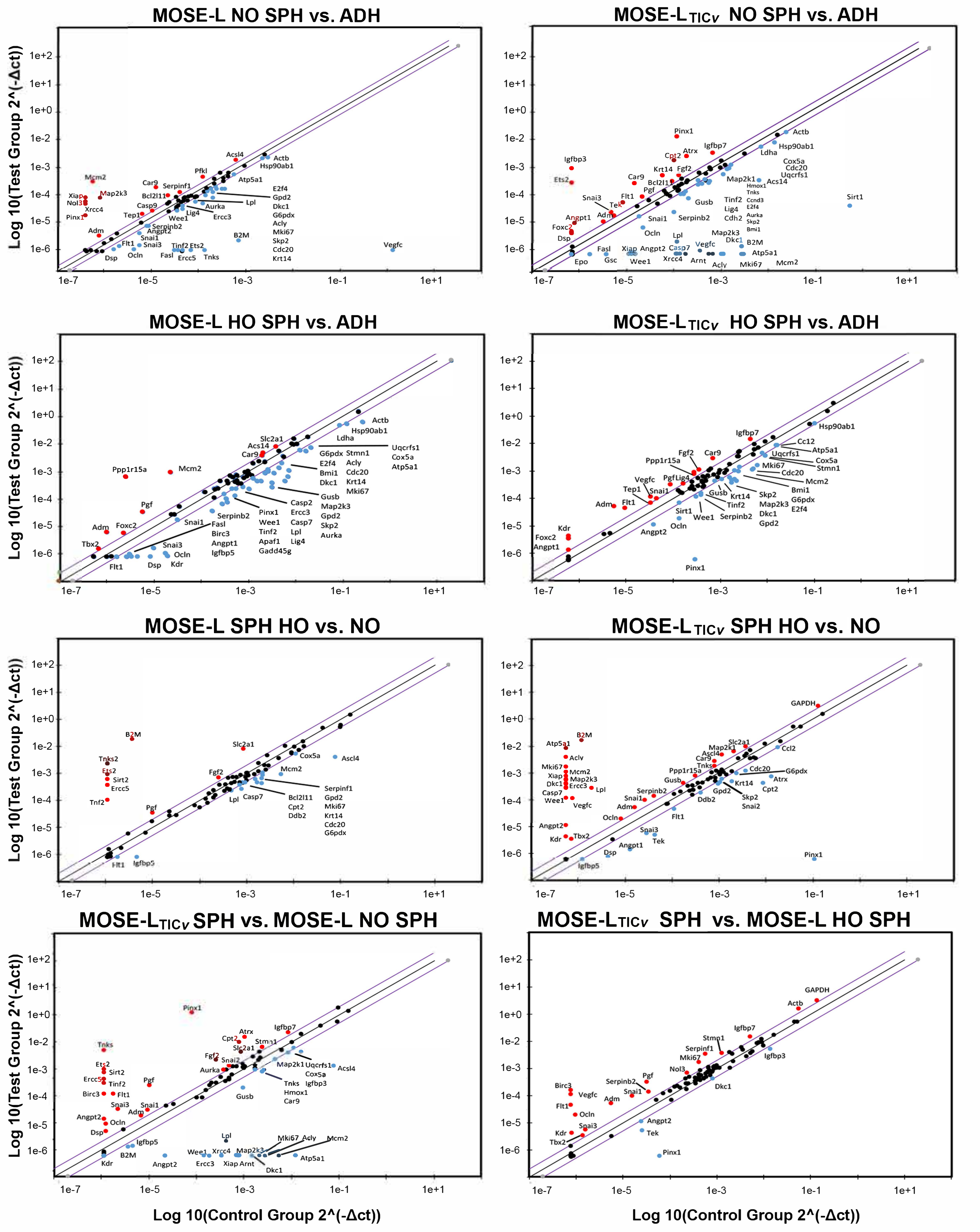

Supplemental Figure S2

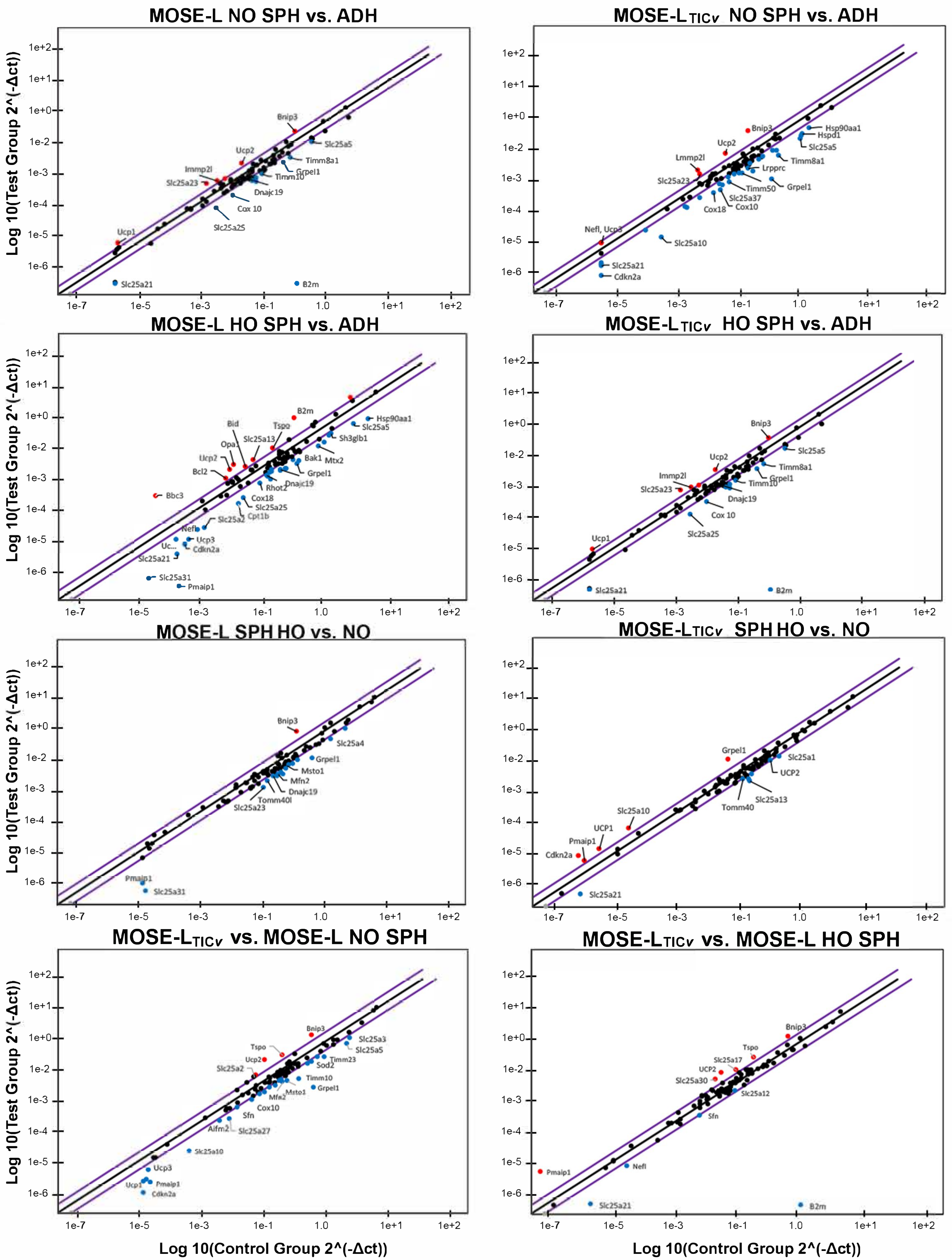

Supplemental Figure S3

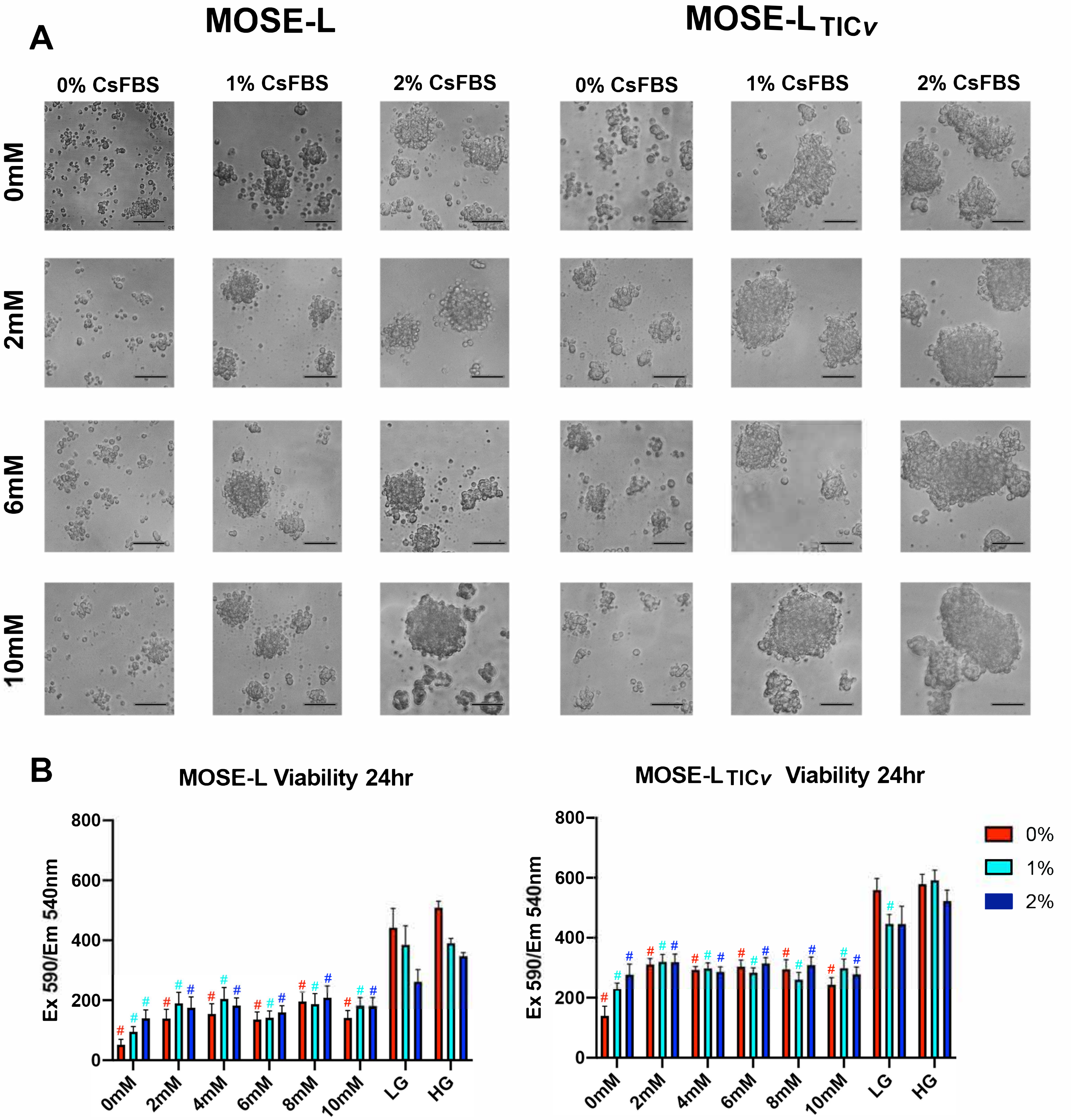

Table S1A: ΔCT for adherent cells						
	Normoxia			Hypoxia		
	MOSE-E	MOSE-L	MOSE-L$_{TICv}$	MOSE-E	MOSE-L	MOSE-L$_{TICv}$
Acly	-2.18	-2.89	-3.04	-2.33	-2.43	-2.41
Acsl4	-1.09	-4.87	-6.32	-0.91	-0.98	-1.37
Adm	9.43	9.04	7.08	2.37	9.94	7.43
Angpt1	6.66	10.42	9.53	7.13	8.55	10.67
Angpt2	5.63	4.18	4.30	5.63	5.16	4.65
Apaf1	1.37	1.17	1.63	1.05	1.65	2.12
Arnt	0.74	0.33	0.75	0.83	0.88	1.51
Atp5a1	-3.65	-4.76	-4.85	-3.62	-4.41	-4.09
Atrx	-0.66	-0.26	-0.11	-0.70	-0.07	0.45
Aurka	-0.35	-1.53	-2.02	-0.43	-1.69	-1.11
Bcl2l11	0.98	2.03	1.16	1.40	1.95	1.76
Birc3	2.93	9.34	2.44	2.48	8.18	2.65
Bmi1	-1.01	-1.59	-1.69	-0.65	-1.43	-1.07
Car9	4.12	3.20	4.43	0.33	-1.08	0.47
Casp2	1.51	1.04	0.66	1.17	1.11	0.98
Casp7	0.92	0.48	0.52	1.17	0.35	1.17
Casp9	3.01	3.61	3.22	2.74	3.04	2.90
Ccl2	-5.27	-4.19	-5.10	-4.81	-3.42	-4.03
Ccnd2	-3.65	-3.59	-2.94	-4.23	-3.09	-2.76
Ccnd3	0.53	0.08	-1.11	-0.24	0.08	-0.49
Cdc20	-2.54	-3.47	-3.71	-2.59	-2.90	-2.39
Cdh2	0.17	0.41	0.02	-0.22	0.87	1.26
Cflar	0.46	1.03	0.94	0.17	0.82	1.02
Cox5a	-3.35	-4.02	-3.88	-3.16	-4.08	-3.28
Cpt2	1.20	1.16	1.02	1.00	1.46	1.90
Ddb2	2.07	1.93	1.88	2.07	2.34	2.78
Dkc1	-0.21	-2.53	-2.32	-0.11	-1.86	-1.25
Dsp	3.83	7.50	9.79	3.13	6.84	10.67
E2f4	-0.27	-1.82	-1.67	-0.65	-1.40	-1.45
Epo	10.48	10.42	9.79	10.18	9.62	10.67
Ercc3	1.38	0.48	0.01	1.16	0.81	1.12
Ercc5	0.89	0.56	0.70	1.17	1.41	1.51
Ets2	0.13	-0.30	9.79	-0.12	0.16	0.57
Fasl	10.48	1.34	8.20	10.18	8.33	10.67
Fgf2	-0.53	1.18	0.61	0.84	1.27	1.42
Flt1	5.08	6.98	5.38	3.82	9.25	6.70
Foxc2	4.26	8.63	9.79	3.59	8.74	10.67
G6pdx	-1.25	-2.41	-1.72	-1.26	-1.23	-1.36
Gadd45g	-1.41	1.17	1.35	-2.32	0.92	1.84
Gpd2	-0.67	-1.99	-1.94	-0.62	-0.93	-0.85
Gsc	9.26	9.84	6.84	9.77	9.94	10.67
Hmox1	-0.72	-1.88	-1.08	-0.85	-0.40	0.73
Igfbp3	-2.61	-3.35	9.79	-0.61	-3.24	-1.42
Igfbp5	5.47	7.92	9.79	8.30	7.76	10.67
Igfbp7	-4.97	-2.66	-2.29	-5.01	-2.19	-2.17
Ing1	-0.50	-0.87	0.39	-0.84	-0.29	-0.33
Kdr	5.40	8.76	9.79	6.13	5.67	10.67
Krt14	-2.94	-3.78	1.98	-3.98	-2.81	-0.15

Ldha	-4.48	-5.70	-6.50	-6.39	-6.46	-6.37
Lig4	1.00	0.54	0.20	1.23	0.48	2.58
Lpl	-0.75	-0.79	0.76	-0.43	-0.06	1.94
Map2k1	-1.03	-2.60	-3.20	-2.32	-2.50	-2.42
Map2k3	-0.90	8.97	-2.00	-0.78	-0.65	-0.72
Mcm2	-1.80	9.66	-4.96	-1.91	5.46	-2.33
Mki67	-2.52	-2.93	-3.21	-2.61	-2.62	-2.69
Nol3	2.52	10.42	0.34	2.32	2.14	0.83
Ocln	10.48	5.45	3.64	10.18	5.79	2.82
Pfkl	-1.01	-1.52	-3.00	-2.98	-3.24	-3.22
Pgf	4.35	7.30	3.72	2.16	7.41	3.49
Pinx1	1.70	10.42	0.73	2.16	1.83	1.70
Ppp1r15a	2.79	2.16	1.74	2.03	8.57	1.75
Serpinb2	7.71	3.98	1.02	7.96	2.53	1.30
Serpinf1	1.58	0.78	-1.13	1.32	0.30	-1.30
Sirt1	1.06	1.92	-14.11	2.55	2.39	2.85
Sirt2	0.61	0.68	0.63	0.65	1.10	1.21
Skp2	0.06	-2.56	-2.09	0.21	-1.70	-0.85
Slc2a1	-0.61	-0.69	-2.09	-2.93	-1.95	-2.41
Snai1	7.20	4.96	3.99	6.19	4.96	4.47
Snai2	0.46	0.70	-0.13	0.15	0.82	0.16
Snai3	9.84	4.91	6.13	10.18	6.63	7.71
Sox10	10.48	10.42	9.79	10.18	10.85	10.67
Stmn1	-2.76	-2.29	-3.40	-2.73	-2.20	-3.24
Tbx2	7.10	10.42	9.79	6.52	10.46	10.67
Tek	8.24	4.70	6.37	8.04	5.36	8.17
Tep1	3.65	4.61	4.17	3.17	4.83	4.83
Tinf2	1.28	1.00	0.44	1.23	1.51	1.28
Tnks	-2.33	-1.66	-1.45	-2.15	-0.76	-0.71
Tnks2	-1.68	-1.76	-2.03	-1.78	-1.18	-1.25
Uqcrfs1	-2.64	-3.54	-3.61	-2.45	-3.68	-3.06
Vegfc	6.62	-20.93	-1.27	7.27	10.85	4.87
Wee1	1.46	1.09	4.67	1.47	1.45	1.66
Xiap	-0.29	10.42	4.89	-0.05	-0.05	0.00
Xrcc4	1.87	10.42	0.81	1.47	0.68	1.16
Actb	-7.49	-8.21	-8.61	-7.50	-8.06	-7.65
B2M	-4.78	-5.22	-4.82	-4.65	-4.19	-3.84
GAPDH	-6.56	-7.77	-7.82	-7.95	-7.76	-8.07
Gusb	0.20	-0.77	-0.22	0.25	-0.37	0.29
Hsp90ab1	-5.67	-7.65	-7.66	-5.89	-6.98	-6.76

Table S1B: ΔCT for homogenous and heterogenous spheroids

	Normoxia					Hypoxia				
	SVF	MOSE-L	MOSE-L +SVF	MOSE-L$_{TICv}$	MOSE-L$_{TICv}$ +SVF	SVF	MOSE-L	MOSE-L +SVF	MOSE-L$_{TICv}$	MOSE-L$_{TICv}$ +SVF
Acly	-3.37	-1.54	-2.64	10.79	-2.70	-2.84	-0.90	-2.41	-1.89	-2.36
Acsl4	-2.04	-6.31	-2.11	-0.25	-2.99	-2.44	-1.89	-2.21	-2.18	-2.06
Adm	1.15	7.17	5.18	5.88	4.65	1.05	7.41	4.96	4.39	4.83
Angpt1	5.13	9.77	6.9	6.24	8.56	4.69	10.29	8.83	9.62	9.48
Angpt2	-0.07	5.47	2.04	10.79	3.78	0.24	5.28	3.93	6.55	3.78
Apaf1	0.91	2.24	1.55	1.78	2.24	1.58	2.79	2.15	2.16	2.25
Arnt	-0.27	0.26	0.03	10.79	0.11	0.31	1.25	1.16	0.95	0.88
Atp5a1	-2.63	-3.68	-3.4	10.79	-3.27	-2.26	-2.82	-3.09	-3.02	-3.05
Atrx	-0.25	-0.11	-0.54	-3.75	-0.28	0.43	0.34	-0.55	0.51	-0.71
Aurka	5.67	1.40	1.83	0.22	0.72	6.25	1.97	1.47	1.13	1.56
Bcl2l11	1.36	0.06	1.05	-0.07	0.69	1.35	1.19	-0.18	0.27	-0.51
Birc3	0.69	9.77	3.47	3.16	2.38	-0.23	10.29	2.41	2.71	1.89
Bmi1	-0.09	-0.80	-0.74	-0.38	-0.68	0.16	-0.16	-0.83	0.02	9.96
Car9	2.66	-1.48	1.42	0.28	0.4	-0.67	-2.14	-2.19	-1.41	-1.82
Casp2	1.85	1.58	1.17	1.12	0.88	1.92	2.20	1.66	1.28	1.70
Casp7	3.20	0.33	1.33	10.79	1.70	3.08	2.01	1.90	1.94	2.15
Casp9	2.11	2.65	2.10	2.68	3.2	2.49	2.59	2.31	2.42	2.24
Ccl2	-6.11	-4.07	-4.72	-4.20	-4.69	-7.48	-3.26	-5.21	-3.05	-5.18
Ccnd2	-2.17	-2.68	-3.17	-2.23	-3.16	-2.6	-3.27	-4.35	-2.74	-4.64
Ccnd3	0.06	0.77	-0.02	-0.18	0.97	0.20	0.58	0.29	-0.04	0.37
Cdc20	3.56	-1.16	-0.44	-1.94	-0.61	4.44	-0.01	-0.30	-0.18	-0.12
Cdh2	3.31	1.00	1.03	1.42	1.57	3.95	1.28	1.48	1.31	1.84
Cflar	-0.97	0.85	-0.45	0.74	-0.04	-0.81	1.11	-0.07	0.85	-0.21
Cox5a	-2.70	-3.52	-3.54	-2.48	-2.87	-2.26	-2.35	-2.86	-1.82	-0.61
Cpt2	1.43	0.27	1.17	-3.15	1.48	1.18	1.21	0.93	1.35	0.92
Ddb2	0.42	1.03	0.46	1.22	1.31	0.65	2.18	1.27	2.59	1.33
Dkc1	2.12	-0.61	-0.08	10.79	-0.18	2.04	0.22	0.25	1.34	0.21
Dsp	6.89	9.63	8.94	7.78	9.49	9.5	10.29	8.73	10.43	9.96
E2f4	0.31	-0.05	0.74	-0.22	-0.11	0.45	0.03	0.57	-0.34	0.80
Epo	9.19	9.77	10.04	10.79	9.73	7.91	10.29	9.20	10.81	9.14
Ercc3	1.33	2.38	1.01	10.79	1.43	1.30	2.19	1.03	1.86	1.99
Ercc5	1.61	9.77	1.15	1.32	1.77	1.95	1.50	0.97	1.86	0.95
Ets2	-0.20	9.77	-1.09	0.13	-0.88	-0.37	0.15	-0.46	-0.15	-0.38
Fasl	9.89	9.72	9.03	10.79	10.38	9.50	10.06	9.97	10.81	9.44
Fgf2	-1.83	1.91	-0.30	-0.96	10.84	-1.53	0.61	-0.61	-0.09	-0.70
Flt1	2.87	9.06	4.94	3.15	6.16	3.10	10.29	4.53	4.56	4.39
Foxc2	2.61	8.4	3.77	7.52	5.66	2.73	7.44	4.92	8.30	4.88
G6pdx	-1.21	-1.18	-1.41	-1.32	-1.33	-0.79	0.25	-0.32	0.09	-0.22
Gadd45g	0.52	2.38	2.16	2.08	2.09	0.87	3.00	0.59	2.34	0.85
Gpd2	0.86	-0.76	-0.62	-0.01	-0.39	0.06	1.17	-0.27	1.47	-0.17
Gsc	5.49	9.77	7.66	10.79	10.4	6.56	10.29	5.82	10.81	9.82
Hmox1	-3.16	-1.33	-1.50	0.47	-0.41	-2.60	-0.95	-0.77	0.04	-0.30
Igfbp3	-5.07	-4.05	-5.18	-1.98	-4.52	-4.91	-3.84	-4.88	-2.25	-4.85
Igfbp5	-3.86	7.70	-2.50	9.58	-0.88	-3.33	10.29	-1.61	10.81	-2.68
Igfbp7	-6.71	-3.12	-4.66	-4.34	-4.64	-6.10	-2.39	-4.94	-3.81	-4.42
Ing1	-0.36	0.06	0.02	-0.53	0.65	-0.39	0.54	-0.25	0.25	-0.33

Kdr	-0.27	9.77	1.37	10.79	3.19	-0.36	10.24	2.69	7.96	2.83
Krt14	9.23	-1.09	-0.48	-0.95	3.67	9.50	0.70	1.21	1.11	2.18
Ldha	-5.16	-5.38	-4.45	-5.25	-4.62	-5.52	-5.5	-5.68	-5.63	-5.48
Lig4	1.14	1.90	0.52	1.80	0.25	0.9	1.49	0.62	1.66	0.47
Lpl	-4.22	1.17	-2.45	8.96	-1.21	-3.59	2.13	-1.35	1.95	-1.26
Map2k1	-2.83	-2.26	-2.20	-1.09	-2.77	-2.78	-1.73	-2.95	-2.58	-2.89
Map2k3	-0.57	0.45	-0.07	10.79	-0.26	-0.32	1.19	-0.10	0.87	0.16
Mcm2	2.07	-2.48	-0.92	10.79	-1.24	3.15	0.16	-0.34	-0.06	-0.06
Mki67	3.73	-1.11	0.21	10.79	-0.28	5.22	1.25	-0.06	-0.65	-0.09
Nol3	1.24	1.20	1.27	0.23	0.43	1.55	2.08	1.32	0.66	1.5
Ocln	9.89	9.58	6.83	6.89	7.06	9.19	9.93	6.4	5.78	7.22
Pfkl	-2.79	-3.36	-2.27	-3.25	-2.2	-2.86	-3.14	-3	-3.54	-3.05
Pgf	-0.27	6.57	1.82	2.18	3.01	-0.97	4.94	0.81	1.73	0.73
Pinx1	2.65	3.62	2.83	-6.78	2.14	2.76	3.99	2.82	10.81	2.63
Ppp1r15a	0.92	1.29	0.4	1.64	-1.68	0.85	0.69	0.2	0.45	0.05
Serpinb2	-2.00	5.45	0.59	4.56	-0.16	-4.72	4.8	-2.91	2.94	-2.9
Serpinf1	-5.71	-0.64	-4.11	-1.39	-3.8	-5.38	0.8	-3.7	-1.68	-3.58
Sirt1	0.91	2.86	1.04	3.61	1.65	0.85	3.19	2.05	3.97	1.8
Sirt2	0.27	9.77	0.42	0.55	0.75	0.61	0.78	0.9	0.73	0.91
Skp2	2.87	0.47	0.45	-0.14	0.32	3.1	1.45	0.94	1.22	0.97
Slc2a1	-3.26	0.17	-1.54	-1.96	-2.27	-3.43	-2.97	-2.85	-3.16	-2.92
Snai1	3.80	6.69	4.48	5.21	4.18	4.15	5.93	4.95	3.43	5.22
Snai2	-1.75	0.99	-2.14	-0.23	-3.47	-1.05	0.49	-2.05	0.84	-2.35
Snai3	9.89	8.79	10.04	5.03	7.63	9.5	9.27	9.97	7.61	9.96
Sox10	5.89	9.77	6.13	10.79	10.84	6.91	10.29	9.97	10.81	9.96
Stmn1	1.25	-1.34	-1.31	-2.62	-1.19	1.57	-0.37	-0.93	-1.74	-1.02
Tbx2	6.05	9.77	7.91	10.40	8.52	6.46	9.41	8.39	8.25	9.96
Tek	2.55	4.89	3.54	4.47	4.8	2.24	5.26	4.38	7.72	4.7
Tep1	1.56	3.31	1.35	3.63	10.03	1.98	4.29	3.70	3.88	3.4
Tinf2	2.17	9.77	2.25	1.85	1.43	2.37	3.34	2.21	2.77	2.31
Tnks	-2.17	-0.83	-2.03	0.26	-2.02	-1.52	-1.15	-2.21	-0.8	-0.55
Tnks2	-1.79	9.77	-1.9	-2.22	-2.18	-1.13	-1.09	-1.82	-1.74	-1.95
Uqcrfs1	-1.86	-3.18	-2.59	-1.84	-2.28	-1.64	-2.52	-2.15	-2.07	-1.9
Vegfc	0.92	9.77	3.31	10.32	2.07	0.8	10.29	1.46	3.21	1.54
Wee1	3.61	2.72	2.72	10.79	2.74	5.19	3.74	3.17	3.16	3.26
Xiap	-0.52	0.39	-0.61	10.79	0.12	-0.26	0.28	-0.65	0.39	-0.86
Xrcc4	0.98	1.49	0.01	10.79	0.45	0.7	1.48	1.32	1.47	1.3
Actb	-5.98	-6.67	-6.98	-7.36	-7.24	-5.94	-5.85	-6.72	-7.16	-6.71
B2M	-6.29	8.05	-5.17	9.66	-4.57	-5.96	-4.12	-4.95	-3.99	-4.65
GAPDH	-6.41	-7.31	-6.64	-7.04	-7.8	-6.66	-7.13	-7.1	-8.17	-7.09
Gusb	-1.19	0.00	-0.43	2.49	0.26	-0.55	0.9	0.66	1.32	0.83
Hsp90ab1	-4.24	-6.57	-5.87	-5.79	-5.87	-4.39	-5.64	-5.26	-5.65	-5.35

	Adherent cells						Spheroids			
	Normoxia			Hypoxia			Normoxia		Hypoxia	
	MOSE-E	MOSE-L	MOSE-TICv	MOSE-E	MOSE-L	MOSE-TICv	MOSE-L	MOSE-TICv	MOSE-L	MOSE-TICv
Aifm2	11.96	12.42	12.38	13.59	12.65	12.83	11.41	12.83	12.27	12.60
Aip	6.40	6.20	6.20	7.98	7.06	7.14	5.71	6.34	6.68	7.1
Bak1	6.48	6.45	5.98	7.95	5.59	6.68	6.49	7.285	7.64	8.08
Bbc3	12.11	14.92	11.19	12.74	16.11	11.53	11.04	11.65	11.63	12.20
Bcl2	9.77	11.53	8.80	10.88	10.97	9.10	9.373	8.53	9.65	9.65
Bcl2l1	6.55	6.39	5.75	7.84	6.87	6.53	6.80	7.18	6.81	7.77
Bid	8.44	8.44	8.17	10.11	9.57	8.75	8.15	9.66	8.34	9.21
Bnip3	7.36	9.04	5.91	4.87	4.59	4.09	4.73	3.11	3.73	2.88
Cdkn2a		15.21			14.00	18.50	7.90	20.38	17.03	17.65
Cox10	9.48	8.41	8.16	10.80	10.47	9.12	9.04	10.46	10.19	10.88
Cox18	9.37	8.91	8.67	11.04	8.51	9.60	10.12	10.79	10.18	10.46
Cpt1b	12.15	11.27	11.02	13.63	10.07	12.53	12.53	12.46	12.46	12.58
Cpt2	8.74	8.78	8.50	10.08	8.73	9.27	7.47	8.46	8.31	8.96
Dnajc19	8.22	7.46	6.69	9.65	6.95	7.55	7.14	7.99	8.79	9.09
Dnm11	5.75	5.7	5.41	7.08	6.39	6.02	6.28	6.31	7.22	7.09
Fis1	6.25	6.78	5.82	7.72	6.70	6.59	6.13	6.87	7.35	7.81
Timm10b	5.96	5.48	5.05	7.42	6.47	5.71	6.00	6.27	7.36	6.89
Grpel1	5.69	4.79	4.01	7.13	5.87	5.0	4.55	9.04	6.71	6.87
Hsp90aa1	1.17	0.23	0.99	2.29	0.57	1.65	2.03	2.77	2.93	2.96
Hspd1	2.79	2.17	1.59	4.30	4.58	2.73	2.89	3.66	3.90	3.77
Immp1l	7.28	6.60	6.03	8.71	6.06	6.59	6.28	6.65	7.54	7.49
Immp2l	10.43	8.09	9.98	11.42	6.66	10.42	7.80	7.98	8.56	9.08
Lrpprc	7.90	7.32	5.93	9.42	7.38	6.92	6.64	7.73	7.82	7.95
Mfn1	7.02	6.64	6.81	8.45	7.09	7.83	7.20	7.32	7.98	8.53
Mfn2	7.57	7.46	7.04	8.57	7.80	7.65	6.83	8.48	8.29	8.70
Mipep	9.09	9.07	8.44	10.35	10.47	9.00	8.88	9.25	10.05	9.62
Msto1	7.65	6.58	6.66	9.29	6.85	8.09	6.47	8.27	7.97	8.90
Mtx2	5.47	4.54	4.80	7.12	4.21	5.75	5.11	5.66	6.00	6.49
Nefl	22.64	14.16			13.06	18.34	7.54	17.04	15.47	17.49
Opa1	7.78	7.99	7.83	8.98	10.40	8.35	7.84	7.81	8.16	8.56
Pmaip1	8.37	16.54		10.42	14.38		8.18	8.83	22.77	18.16
Rhot1	7.63	7.14	7.06	9.06	7.055	7.79	7.06	7.89	7.48	7.88
Rhot2	9.20	8.97	8.08	10.63	8.05	9.25	8.49	9.07	8.96	9.20

Sfn	10.87	10.05	10.56	11.38	9.34	11.26	10.15	11.39	10.20	11.75
Sh3glb1	3.71	3.3	3.80	5.03	3.18	4.68	3.87	4.04	4.10	4.92
Slc25a1	5.77	6.20	5.12	6.99	5.88	5.87	5.92	5.32	6.73	6.59
Slc25a10	13.93	12.99	12.85	14.19	12.50	14.65	13.58	16.25	13.27	14.59
Slc25a12	7.61	7.13	7.39	9.16	8.95	8.747	7.00	7.52	7.59	9.04
Slc25a13	5.90	5.87	5.96	7.27	7.33	7.23	6.94	7.47	8.16	9.25
Slc25a14	9.31	9.99	9.64	10.79	9.89	9.80	9.77	9.84	10.65	10.62
Slc25a15	8.89	7.52	7.99	10.20	7.68	9.18	8.51	9.87	9.71	9.91
Slc25a16	7.84	8.25	8.89	9.52	7.63	9.75	8.78	8.85	8.66	8.84
Slc25a17	6.91	6.50	5.62	8.39	6.81	6.40	6.54	6.37	7.52	6.64
Slc25a19	9.09	9.17	9.13	10.47	10.83	10.06	9.02	9.97	10.24	10.10
Slc25a2	15.06	14.50	14.14	15.87	12.53	15.16	15.31	15.51	15.18	15.19
Slc25a20	8.80	9.04	8.89	10.59	9.03	9.89	7.79	9.09	8.76	9.59
Slc25a21		16.08			14.51	19.40	9.17	19.71	18.11	21.83
Slc25a22	11.34	11.35	10.59	12.35	11.30	11.53	10.75	11.40	11.77	11.44
Slc25a23	10.07	10.31	9.77	12.42	10.55	11.28	8.14	8.51	10.09	9.51
Slc25a24	7.67	8.54	6.81	8.75	7.90	7.37	8.73	7.67	9.37	8.80
Slc25a25	9.73	9.83	9.80	11.00	9.71	10.49	10.91	11.42	11.81	12.46
Slc25a27	12.05	11.50	10.86	13.37	10.93	12.47	10.68	12.61	11.63	12.68
Slc25a3	2.40	1.75	1.53	4.10	2.89	2.67	1.90	3.51	2.54	3.89
Slc25a30	8.58	8.44	6.99	9.56	7.76	7.54	8.93	7.93	9.00	7.69
Slc25a31		17.34			16.89		9.24	10.44	20.86	22.03
Slc25a37	8.89	10.00	7.41	10.59	10.01	8.51	10.11	9.42	9.81	9.44
Slc25a4	3.46	3.45	3.33	5.14	3.29	4.39	3.17	4.14	4.59	4.60
Slc25a5	1.54	0.53	1.69	3.395	1.62	2.78	2.14	4.09	3.48	4.26
SOD1	5.03	5.91	3.89	6.788	6.32	5.36	4.27	5.57	5.35	6.13
SoOD2	6.01	5.66	4.76	8.18	5.03	5.80	4.99	6.38	5.60	6.00
Stard3	10.13	10.62	9.78	11.46	10.16	10.62	9.36	9.71	9.66	10.46
Taz	8.91	9.01	9.06	10.27	9.12	10.13	8.62	9.60	8.79	9.51
Timm10	7.58	6.10	5.54	9.32	7.08	6.73	5.60	8.1	6.99	8.27
Timm17a	6.29	5.56	5.61	7.98	6.11	6.65	6.49	6.95	7.24	7.50
Timm17b	7.40	7.53	7.01	8.83	7.87	7.58	6.67	7.21	8.60	8.04
Timm22	7.61	6.46	6.44	9.32	5.77	7.53	7.26	7.47	8.01	8.03
Timm23	4.02	2.82	3.637	5.95	3.40	4.63	3.80	5.58	4.86	5.76
Timm44	7.88	7.46	7.07	9.46	7.86	7.86	6.93	8.23	8.02	8.56
Timm50	7.30	6.48	6.37	8.55	6.99	7.23	7.07	8.33	8.61	8.70
Timm8a1	4.97	2.97	3.46	6.61	3.74	4.50	4.73	6.15	5.63	6.20
Timm8b	4.88	3.78	4.24	6.60	4.14	5.11	4.06	4.93	5.25	5.62

Timm9	7.74	5.77	6.26	9.47	5.55	7.47	7.03	7.04	6.65	7.03
Tomm20	4.73	4.03	3.70	6.63	4.45	4.84	3.65	4.24	3.46	4.26
Tomm22	6.44	5.53	4.69	8.16	6.47	5.71	5.93	6.30	6.86	6.59
Tomm34	6.57	6.03	6.29	8.22	5.65	6.81	6.40	6.06	6.53	6.55
Tomm40	8.18	7.45	6.62	9.50	7.95	7.74	7.80	8.41	9.31	8.93
Tomm40l	8.09	7.07	7.02	9.77	7.59	8.31	7.41	7.70	8.82	8.92
Tomm70a	6.25	5.24	5.02	8.20	6.06	6.11	5.73	6.64	6.40	7.00
Trp53	6.73	6.58	5.83	8.61	6.64	6.84	6.64	7.36	7.80	7.97
Tspo	6.29	7.65	5.52	7.45	7.56	5.69	6.87	5.44	6.26	5.18
UCP1	16.43	15.80			14.59	18.43	8.02	18.40	16.48	16.85
UCP2	6.66	9.10	7.73	8.69	10.70	8.45	8.13	5.96	8.61	6.96
UCP3	17.89	14.43	18.20		13.68	15.72	17.98	17.02	16.47	16.92
Uxt	7.50	7.50	8.05	9.17	6.52	7.86	7.83	8.38	8.25	8.88

Table S2: Changes in the gene expression (ΔCT values) of mitochondrial regulators in response to aggregation and hypoxia
